# Stalling of the endometrial decidual reaction determines the recurrence risk of miscarriage

**DOI:** 10.1126/sciadv.adv1988

**Published:** 2025-06-25

**Authors:** Joanne Muter, Chow-Seng Kong, Mireia Taus Nebot, Maria Tryfonos, Pavle Vrljicak, Paul J. Brighton, Danai B. Dimakou, Megan Vickers, Hiroyuki Yoshihara, Sascha Ott, Bee K. Tan, Phillip R. Bennett, Siobhan Quenby, Alex Richter, Hilde Van de Velde, Emma S. Lucas, Thomas M. Rawlings, Jan J. Brosens

**Affiliations:** ^1^ Warwick Medical School, Division of Biomedical Sciences, University of Warwick, CV4 7AL Coventry, UK.; ^2^ Tommy’s National Centre for Miscarriage Research, University Hospitals Coventry & Warwickshire National Health Service Trust, CV2 2DX Coventry, UK.; ^3^ Research Group Genetics, Reproduction and Development, Vrije Universiteit Brussel, 1050 Brussels, Belgium.; ^4^ Department of Clinical Immunology Service, University of Birmingham, B15 2TT Birmingham, UK.; ^5^ Department of Cardiovascular Sciences, University of Leicester, LE1 7RH Leicester, UK.; ^6^ Tommy’s National Centre for Miscarriage Research, Imperial College London, W12 0HS London, UK.

## Abstract

In every menstrual cycle, progesterone acting on estrogen-primed endometrium elicits an inflammatory decidual reaction, rendering it poised for embryo implantation and transformation into the decidua of pregnancy. Here, we show that the sequential functions of the decidual reaction—implantation and decidualization—pivot on the time-sensitive loss of progesterone-resistant *DIO2*^+^ stromal cells that form a specialized implantation niche and reciprocal expansion of progesterone-dependent *PLA2G2A*^+^ predecidual cells. Simultaneously, uterine natural killer (uNK) cell proliferation results in the accumulation of immunotolerant subsets. Examination of endometrial biopsies from 924 women revealed that the recurrence risk of miscarriage closely aligns with the incidence of a weakened or stalled decidual reaction, more so than poor uNK cell expansion. Analysis of paired biopsies obtained in different cycles and modeling in assembloids intimated that prior miscarriages disrupt intercycle endometrial homeostasis and calibration of the decidual reaction. Our findings show that erosion of the decidual reaction following a miscarriage drives the recurrence risk irrespective of maternal age.

## Teaser

The frequency of menstrual cycles culminating in a suboptimal decidual reaction determines the recurrence risk of miscarriage.

## INTRODUCTION

Miscarriage denotes the loss of a pregnancy before viability (*1*). Approximately one in three embryos perishes following implantation in healthy women, although often before routine detection of pregnancy (*2*, *3*). This attrition rate reflects the high prevalence of chromosomal errors in preimplantation human embryos (*4*) and the physiological role of the endometrium in selecting against low-fitness embryos (*2*, *3*, *5*, *6*). The pooled miscarriage risk in all clinically recognized pregnancies is an estimated 15% (*1*), with most losses (~90%) occurring before the onset of uteroplacental perfusion at the end of the first trimester (*7*). Epidemiological studies consistently highlight that two factors, maternal age and the number of preceding pregnancy losses, disproportionally affect miscarriage rates (*7*–*9*). The age-dependent risk reflects the increase in aneuploid pregnancies in women aged 35 years and older, mirroring the incidence of meiotic chromosome errors in oocytes and embryos (*10*, *11*). Each prior pregnancy loss further compounds the risk stepwise by 5 to 10% (*7*–*9*), but the underlying mechanism is unknown. A plausible but untested hypothesis is that the recurrence risk of miscarriage reflects the frequency of menstrual cycles culminating in an endometrial environment permissive of embryo implantation but inadequately prepared for decidual transformation (*3*), that is, the formation of a robust immunotolerant matrix that anchors and supports the semiallogenic placenta throughout pregnancy (*2*, *5*).

Each menstrual cycle starts with the shedding of the superficial endometrial layer, bleeding, and reepithelization of the basal layer (*2*). Following menstruation, estradiol-dependent regeneration of the superficial layer, on average, quadruples the thickness and volume of the uterine mucosa before ovulation (*12*). Local morphogen and cytokine gradients regulate epithelial and stromal cell proliferation, resulting in tissue stratification and positional cell specification (*2*). After ovulation, progesterone acting on this spatial template triggers a decidual reaction, an endogenous inflammatory tissue response that heralds the start of the 4-day midluteal implantation window (*2*, *13*). Histologically, the implantation window coincides with the onset of glandular secretion, marked oedema, and proliferative expansion and differentiation of uterine natural killer (uNK) cells (*14*, *15*). A canonical feature of the decidual reaction is the progesterone-dependent reprogramming of stromal cells, termed predecidual cells, under the control of evolutionarily conserved transcription factors (*5*, *16*, *17*). By secreting interleukin-15 (IL-15), predecidual cells activate cytolytic uNK cells to prune stressed and damaged cells, curtailing tissue inflammation (*18*, *19*). The emergence of mature decidual cells, characterized by their epithelioid morphology and stress-resistant phenotype, signals the window’s closure (*5*, *14*).

Implantation in humans is interstitial, meaning that the embryo fully breaches the luminal epithelium and embeds in the underlying stroma (*2*, *20*). Modeling this process in endometrial assembloids, comprising gland-like organoids and primary stromal cells, showed that the decidual reaction imposes a broad binary state on stromal cells, separating progesterone-responsive and progesterone-resistant subsets (*21*). Progesterone-resistant stromal cells, including transitional epithelial–mesenchymal and acutely senescent cells, create a dynamic implantation environment for cocultured embryos. However, their persistence also leads to spontaneous disintegration of assembloids. By contrast, the lack of progesterone-resistant stromal cells accelerates the emergence of decidual cells and entraps embryos in a static matrix, precluding implantation (*21*). These observations suggest that implantation and decidual transformation require time-sensitive rebalancing of functionally distinct stromal subsets across the implantation window. Here, we characterize this process in vivo, explore the interplay between stromal and uNK cell dynamics, and examine whether the frequency of menstrual cycles resulting in a blunted or stalled decidual reaction accounts for the recurrence risk of miscarriage.

## RESULTS

### Characterization of *DIO2*^+^ and *PLA2G2A*^+^ endometrial stromal subsets

**Fig. 1.**
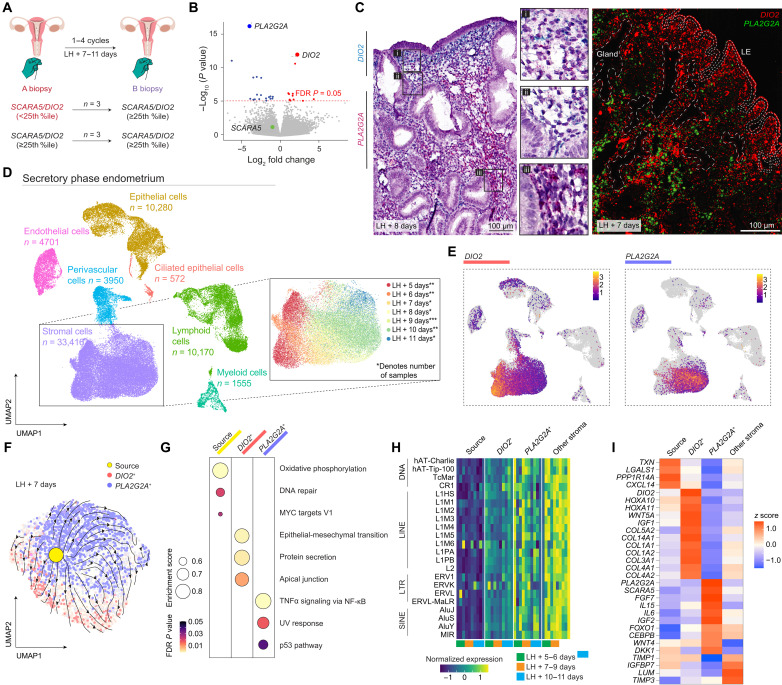
*DIO2*^+^ and *PLA2G2A*^+^ mark functionally distinct stromal subsets. (**A**) Depiction of endometrial sampling in six subjects across two menstrual cycles. For each sample (*n* = 12), the ratio of *SCARA5* and *DIO2* transcripts measured by reverse transcription quantitative polymerase chain reaction was normalized for the day of the biopsy relative to the LH surge; samples were deemed progesterone resistant [*SCARA5/DIO2* < 25th percentile (25th %ile), highlighted in red] or progesterone responsive (*SCARA5/DIO2* ≥ 25th percentile). (**B**) Volcano plot showing gene expression variance between progesterone-resistant and progesterone-responsive endometrial biopsies. Transcripts of interest are highlighted. FDR, false discovery rate. (**C**) Representative images of *DIO2* and *PLA2G2A* transcripts in midluteal endometrium by chromogenic (left) and fluorescent (right) smISH. LE, luminal epithelium. (**D**) Uniform manifold approximation and projection (UMAP) projections of scRNA-seq data showing the cellular composition of luteal phase endometrium (*n* = 12). The color key in the inset depicts days post-LH surge; asterisks denote sample numbers for each day. (**E**) Feature plots showing relative expression of *DIO2* and *PLA2G2A* in different cell types. The color scale represents log-transformed average gene expression. (**F**) RNA velocity stream mapped onto a UMAP plot of stromal subsets from a midluteal biopsy. (**G**) GSEA enrichment plot. The dot size corresponds to the enrichment score; the color key denotes Benjamini-Hochberg–adjusted *P* values. TNFα, tumor necrosis factor–α; NF-κB, nuclear factor κB; UV, ultraviolet. (**H**) Heatmap of normalized TE-derived transcript levels encompassing DNA transposons (DNA), long interspersed nuclear elements (LINEs), long terminal repeats retrotransposons (LTRs), and short interspersed nuclear elements (SINEs). (**I**) Heatmap of *z* score–scaled genes in source, *DIO2*^+^, *PLA2G2A*^+^, and other stromal cells.

We previously reported that *SCARA5* (ferritin receptor) and *DIO2* (iodothyronine deiodinase 2), genes induced and repressed by progesterone, respectively, mark different stromal subsets (*19*). When normalized for the timing of an endometrial sample relative to the preovulatory luteinizing hormone (LH) surge, high endometrial *DIO2* expression mirrors lower *SCARA5* transcript levels and vice versa (*19*), indicating that the ratio of these marker genes is a putative measure of the overall progesterone responsiveness of stromal cells in tissue samples. However, *SCARA5* is expressed across the luteal phase in a relatively narrow dynamic range (*19*), suggesting that it lacks sensitivity to monitor emerging predecidual cells. To explore this further, we measured *SCARA5* and *DIO2* transcripts in 12 endometrial biopsies obtained in different menstrual cycles from six subjects (table S1). Following normalization to the LH surge (*19*), progesterone responsiveness was deemed normal in nine samples (*SCARA5*/*DIO2* ≥ 25th percentile) and impaired in three biopsies (*SCARA5*/*DIO2* < 25th percentile) (Fig. 1A). RNA sequencing (RNA-seq) revealed that high *DIO2* transcript levels in the three biopsies designated as progesterone-resistant were associated with loss of *PLA2G2A*, much more so than *SCARA5* (Fig. 1B and data file S1). *PLA2G2A* encodes the acute-phase protein phospholipase A2 group IIA (PLA2G2A). By hydrolyzing the ester bond of the fatty acyl group attached at the sn-2 position of phospholipids, PLA2G2A promotes the release of arachidonic acid (*22*), the first step in the production of the ancestral decidual signal, prostaglandin E2 (*23*). Multiplexed single-molecule in situ hybridization (smISH) demonstrated that *PLA2G2A* expression marks stromal cells separated from the luminal epithelium by a layer of *DIO2*^+^ cells (Fig. 1C). To characterize the *DIO2*^+^ and *PLA2G2A*^+^ subsets, we performed single-cell RNA-seq (scRNA-seq) on 12 additional biopsies timed to span the implantation window (table S2). Following dimensionality reduction, seven major cell clusters were identified, including 33,416 stromal cells (Fig. 1D, fig. S1A, and data file S1). Expression of *PLA2G2A*, such as *DIO2*, was highly enriched in stromal cells (*P* < 2.3 × 10^−308^; Fig. 1B). Temporal changes in gene expression were evident upon clustering of stromal cells by the day of the biopsy relative to LH surge (LH + 5 to +11 days) (Fig. 1D). Further, the putative implantation window (LH + 6 to +9 days) coincided with a conspicuous shift from a preponderance of *DIO2*^+^ to an abundance of *PLA2G2A*^+^ cells (Fig. 1E and fig. S1C). *DIO2*^+^ and *PLA2G2A*^+^ subsets constituted ~43% of all captured stromal cells, but cells coexpressing both genes were relatively rare (Fig. 1C), averaging at 3.6% (SD, ±2.9%) (fig. S1C). In 10 of 12 samples, RNA velocity analysis inferred that a discrete cluster of source cells drives gene expression in the *DIO2*^+^ and *PLA2G2A*^+^ subsets (Fig. 1F and fig. S1D). Hallmark gene set enrichment analysis (GSEA) predicted heightened oxidative phosphorylation and activation of DNA repair pathways in source cells (Fig. 1G), indicative of acute cellular stress. Notably, source cells and, to a lesser extent, *DIO2*^+^ cells differ from other stromal subsets by the depletion of transcripts derived from transposable elements (TEs) (Fig. 1H), suggesting lack of prior exposure to replication stress (*24*). Visualization of hallmark DNA repair genes on a spatial transcriptomic map of midluteal endometrium identified putative “hotspots” of damaged cells residing some distance from the luminal epithelium (fig. S1E). Decidual cells may have emerged in ancestral eutherians due to progesterone gaining control over the senescence-associated secretory phenotype, converting a proinflammatory cell state into an anti-inflammatory state (*17*). In keeping with this paradigm, the enriched hallmark gene sets in *PLA2G2A*^+^ cells predicted nuclear factor κB and p53 activation (Fig. 1G), two pivotal signaling pathways in cellular senescence (*25*). Transcriptional profiling confirmed that *PLA2G2A*^+^ cells are predecidual cells, exemplified by the expression of genes coding canonical decidual transcription factors (e.g., *FOXO1* and *CEBPB*) and secreted proteins (e.g., *IL15*, *WNT4*, *IGF2*, *FGF7*, and *DKK1*) (Fig. 1I) (*5*). By contrast, *DIO2*^+^ cells are enriched in hallmark epithelial-mesenchymal transition (EMT) genes (Fig. 1G). DIO2 regulates energy expenditure in cells by catalyzing the conversion of prohormone thyroxine (3,5,3′,5′-tetraiodothyronine) to the bioactive thyroid hormone [3,5,3′-triiodothyronine (T3)] (*26*). Subluminal *DIO2*^+^ cells produce an abundance of extracellular matrix (ECM) components (Fig. 1I), plausibly explaining the need for heightened energy expenditure. *DIO2*^+^ cells further express multiple genes that govern Müllerian duct patterning during development, including *WNT5A*, *HOXA10*, and *HOXA11* (Fig. 1I) (*27*). Thus, an embryo breaching the luminal epithelium encounters a specialized stromal microenvironment harboring *DIO2*^+^ cells. The EMT gene signature predicts that subluminal *DIO2*^+^ cells have a migratory and invasive phenotype (*28*), properties considered essential for interstitial implantation (*5*, *6*). Underlying this specialized implantation niche are the precursors of anti-inflammatory decidual cells, which, in pregnancy, cooperate with invasive trophoblast and local immune cells to form the uteroplacental interface (*29*, *30*).

### Spatiotemporal regulation of uNK subsets

**Fig. 2.**
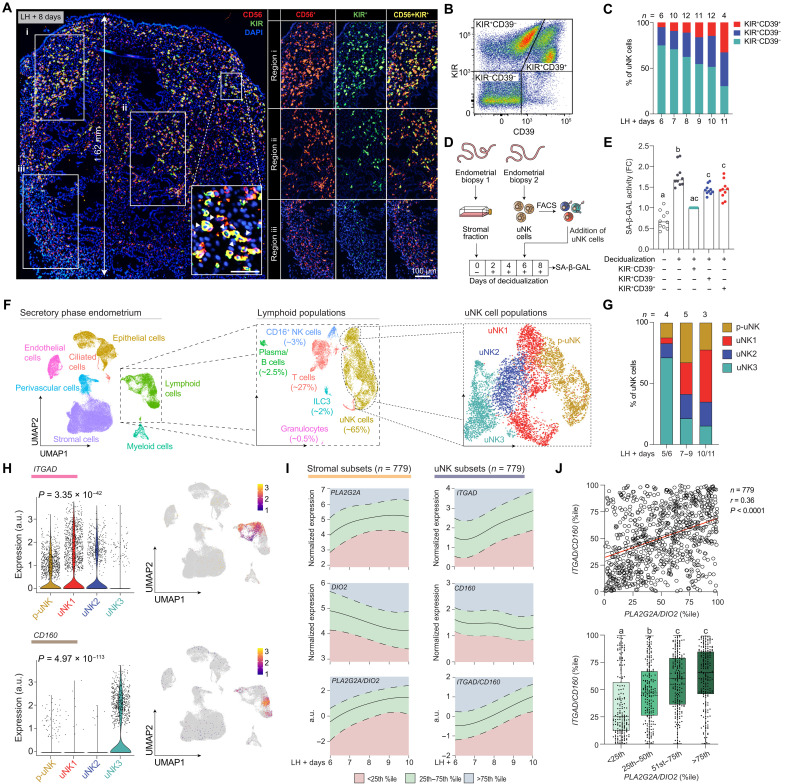
Spatiotemporal dynamics of uNK cells. (**A**) Representative image of CD56 (neural cell adhesion molecule 1) and pan-KIR immunofluorescence in midluteal endometrium. CD56 and pan-KIR immunoreactivity in different regions is shown separately in boxes i to iii. The insert shows a region at higher magnification. Scale bars, 50 μm. Nuclei are stained with 4′,6-diamidino-2-phenylindole (DAPI). (**B**) Flow cytometry density plot showing different uNK subsets based on KIR and CD39 expression. (**C**) Bar graph depicting the temporal changes in the relative abundance of uNK subsets in 55 endometrial samples, as determined by flow cytometry. (**D**) Schematic of the experimental design. The uNK subsets were purified by FACS using pan-KIR and CD39 antibodies. (**E**) Bar graphs showing median fold change (FC) in SA-β-GAL activity in 10 biological repeat experiments. Individual data points are also shown. Different letters indicate statistically significant differences at *P* < 0.01, Friedman test with Dunn’s multiple comparisons test. (**F**) UMAP projections of scRNA-seq data showing lymphoid populations and uNK subsets. (**G**) Bar graphs depicting the temporal changes in the relative abundance of uNK subsets, as determined by scRNA-seq. (**H**) Violin plots (left) and feature plots (right) of *ITGAD* and *CD160* expression with *P* values based on Wilcoxon rank sum test; a.u., arbitrary units. (**I**) Relative expression of stromal and uNK subset marker genes and their ratios in 779 endometrial biopsies obtained 6 to 10 days after the LH surge. The solid line indicates median expression; the dotted lines mark the boundaries of the upper and lower quartiles. Ratios were fitted against a gamma or a log-normal distribution depending on LH + day. (**J**) Top: Spearman’s correlation between *ITGAD/CD160* and *PLA2G2A/DIO2* percentiles. Bottom: Box plots comparing *ITGAD/CD160* percentiles and *PLA2G2A/DIO2* percentiles grouped in quartile bins. Different letters above the whiskers indicate significance between groups at *P* < 0.05, Kruskal-Wallis test with Dunn’s multiple comparisons test.

Accumulation of uNK cells in the endometrium is a consistent feature of the decidual reaction in menstruating primates (*31*). NK cells are effector lymphocytes of the innate immune system involved in the clearance of stressed and damaged cells, allorecognition, and antimicrobial defenses (*32*). In human endometrium, proliferative expansion promotes differentiation of CD56^+^ uNK cells, characterized by the sequential acquisition of killer cell immunoglobulin-like receptors (KIRs) and the ectonucleotidase CD39 (*15*). As local cues regulate tissue-resident NK cells (*15*), we examined the spatial distribution of CD56^+^KIR^−^ and CD56^+^KIR^+^ uNK cells during the implantation window. Mirroring the spatial distribution in early pregnancy (*33*), dual-color fluorescence microscopy revealed an overrepresentation of CD56^+^KIR^+^ uNK cells in stroma abutting the luminal epithelium and CD56^+^KIR^−^ uNK cells in the underlying tissue (Fig. 2A). Flow cytometric analysis of 55 timed biopsies (table S2) confirmed the rapid expansion of KIR^+^CD39^−^ and KIR^+^CD39^+^ uNK subsets as the endometrium cycles across the implantation window (Fig. 2, B and C, and fig. S2A). We reasoned that the spatial organization of phenotypically distinct uNK cells reflects functional adaptation to specific stromal microenvironments. We investigated this possibility by coculturing decidualizing primary stromal cells with CD56^+^ uNK cells separated first by fluorescence-activated cell sorting (FACS) into different subsets based on the expression of KIR and CD39 (Fig. 2D and table S2). Senescence-associated β-galactosidase (SA-β-GAL) activity increases markedly upon decidualization (Fig. 2E), reflecting the emergence of decidual-like senescent cells (*18*, *19*). Cocultured uNK cells attenuated SA-β-GAL activity, with KIR^−^CD39^−^ uNK cells being twice as effective as KIR^+^CD39^−^ or KIR^+^CD39^+^ uNK cells (Fig. 2E). These observations imply that KIR^−^CD39^−^ uNK cells, which reside with *PLA2G2A*^+^ predecidual cells, are cytolytic cells that constrain decidual inflammation through targeted killing of stressed and damaged cells. The crowding of attenuated KIR^+^ uNK cells in the subluminal implantation niche is in keeping with their known roles in pregnancy, including conferring maternal immune tolerance to the semiallogenic conceptus and promoting trophoblast invasion (*29*).

On the basis of scRNA-seq analysis, uNK cells comprise ~65% of lymphoid immune cells in the midluteal endometrium, more than twofold the abundance of T cells (~27%) (Fig. 2F, fig. S2B, and data file S1). Apart from a discrete cluster of peripheral blood CD16^+^ NK cells (~3.6% of lymphoid cells), we identified four uNK subsets (Fig. 2F), in keeping with other studies (*15*, *34*). All *KIR*^+^ uNK subsets (uNK1, uNK2, and proliferating uNK cells) expand rapidly upon opening of the implantation window, thus diluting the relative abundance of uNK3 cells, corresponding to cytolytic KIR^−^CD39^−^ uNK cells (Fig. 2G and fig. S2C). We identified *ITGAD* (integrin subunit alpha D) and *CD160* (CD160 molecule) as genes selectively enriched, respectively, in *KIR*^+^ and *KIR*^−^ uNK subsets (Fig. 2H and fig. S2D). In agreement, the ratio of *ITGAD*/*CD160* transcript levels discriminated between freshly isolated KIR^−^ and KIR^+^ uNK cells (fig. S2E). To gain further insights into the cellular dynamics across the implantation window, we quantified the expression of genes marking the stromal (*PLA2G2A* and *DIO2*) and uNK (*ITGAD* and *CD160*) subsets in 779 timed endometrial samples (table S2). Next, we calculated the ratios of paired marker genes and established percentile ranks (Fig. 2I). This analysis revealed a significant correlation (*r* = 0.36, *P* < 0.0001) between the strength of the decidual reaction in stromal cells, as measured by *PLA2G2A*/*DIO2* percentiles, and the magnitude of uNK cell expansion (*ITGAD*/*CD160* percentiles) (Fig. 2J). However, divergence was apparent in a substantial proportion of samples. Together, the data show that time-sensitive rebalancing of functionally distinct stromal and uNK subsets across the implantation window is a coregulated but not necessarily interdependent process.

### Regulation of stromal and uNK subsets by hormonal and embryonic cues

Next, we investigated how different cues may affect stromal and uNK cell dynamics in midluteal endometrium. We focused first on spatial variations in the endometrium, the relationship between peripheral blood NK and local uNK cells, and the impacts of circulating ovarian hormone levels and local T3 signaling. We then explored the putative contributions of endometrial subsets during implantation and examined the effects of embryonic fitness signals on the decidual reaction and uNK cell expansion.

**Fig. 3.**
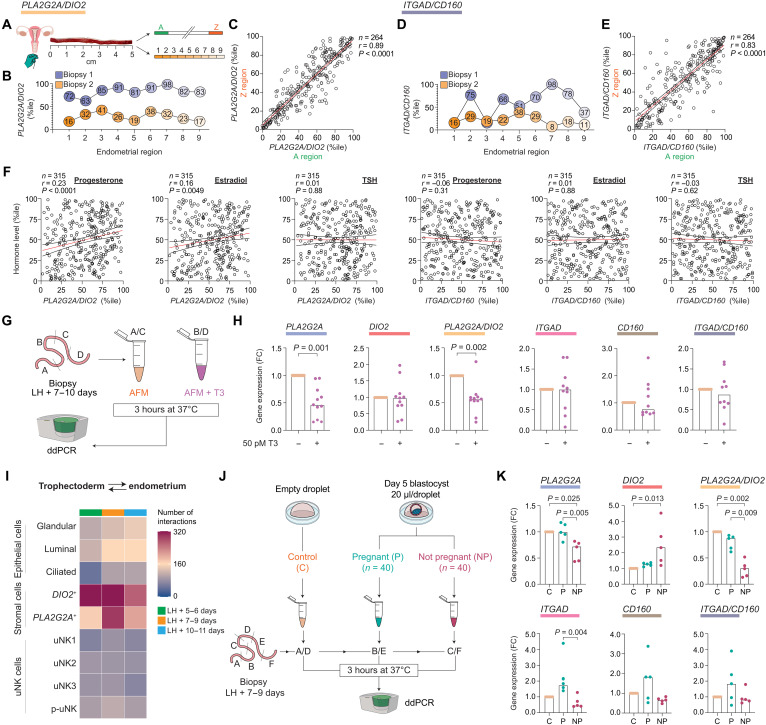
Regulation of stromal and uNK subsets. (**A**) Schematic representation of the spatial analysis of normalized *PLA2G2A/DIO2* and *ITGAD/CD160* ratios (percentiles) in endometrial biopsies. (**B** and **D**) Representative analysis of two independent samples with normalized gene ratios measured across sequential (~0.5 cm) regions. (**C** and **E**) Spearman’s correlation of normalized gene ratios at the opposite ends of the samples (A/Z regions) from 264 subjects. (**F**) Spearman’s correlation between normalized *PLA2G2A/DIO2* or *ITGAD/CD160* ratios (percentiles) and circulating progesterone, estradiol, and TSH levels normalized to the day of sampling post-LH surge (percentiles). Paired endometrial and peripheral blood samples were obtained from 315 subjects. (**G**) Schematic of experimental design. Freshly isolated endometrial samples were divided, and randomly selected sections were incubated for 3 hours in additive-free media (AFM) supplemented or not with 50 pM T3 before gene expression analysis using digital droplet polymerase chain reaction (ddPCR). (**H**) Bar graphs showing median FC in gene expression/gene ratios of 11 and 10 biological repeat experiments for stromal and uNK subsets, respectively. Individual data points are also shown; *P* values are based on the Wilcoxon matched-pairs signed-rank test. (**I**) Heatmap showing the temporal changes in the number of predicted receptor-ligand interactions between uNK, stromal, or epithelial subsets and trophectoderm cells from day 6/7 human blastocysts. (**J**) Schematic of the experimental design. Freshly isolated endometrial samples were divided, and sections were incubated for 3 hours in pooled spent medium, diluted 1:1 in AFM, of IVF blastocysts that resulted in a clinical pregnancy (P) or not [not pregnant (NP)]. Pooled embryo-free droplets served as the control (C) group. (**K**) Bar graphs showing median FC in gene expression/gene ratios of five biological repeat experiments. Individual data points are also shown; *P* values are based on the Friedman test with Dunn’s multiple comparisons test.

Embryos implant preferentially near the uterine fundus (*20*), raising the possibility that spatial differences preclude accurate assessment of the decidual reaction or uNK cell expansion in tissue samples. Pipelle biopsies, which sample the superficial endometrium tangentially at a depth of 1 to 2 mm, can be several centimeters long (Fig. 3A). Analysis of marker genes across the entire length of biopsies or at the opposite ends of the samples (*n* = 264) showed that spatial variability is limited, albeit more pronounced for uNK than stromal subsets (Fig. 3, B to E, and table S2). Next, we measured circulating progesterone, estradiol, and thyroid-stimulating hormone (TSH) levels in 315 subjects (table S2). Progesterone and estradiol levels, normalized to the LH surge (fig. S3A), correlated positively with *PLA2G2A*/*DIO2* but not with *ITGAD*/*CD160* ratios (Fig. 3F), reflecting the direct hormonal dependency of predecidual cells but not uNK cells (*5*, *15*). Although uNK cells originate from circulating progenitor cells (*15*), we observed no correlation between the abundance of peripheral blood NK cells and local uNK cell expansion, as measured by normalized *ITGAD*/*CD160* ratios (fig. S3B). TSH levels had no discernible impact on stromal or uNK subsets (Fig. 3F). To explore the role of local DIO2 activity, we incubated endometrial fragments from freshly obtained midluteal biopsies in additive-free medium supplemented with or without T3 for 3 hours (Fig. 3G, fig. S3C, and table S2). T3 treatment did not affect uNK cell marker genes but, as observed in other tissues (*35*), repressed *PLA2G2A* expression in predecidual cells, thereby reducing the *PLA2G2A*/*DIO2* ratio by ~50% (Fig. 3H). Further, T3 also inhibited other predecidual genes, including *FOXO1*, *SCARA5*, and *IL15* (fig. S3D), indicating that DIO2-dependent T3 production confers progesterone resistance in the subluminal implantation niche.

We used a computational approach to probe how the spatiotemporal changes in endometrial subpopulations could affect embryo implantation. Enumeration of predicted receptor-ligand interactions by CellPhoneDB indicated that the stromal subsets, especially *DIO2*^+^ cells, play an outsized role in the cross-talk between the endometrium and embryonic trophectoderm cells, the precursors of placental cell lineages (Fig. 3I and data file S1). Mining of the data for time-sensitive, subset-specific interactions implicated *DIO2*^+^ cells in decoding trophoblast-derived Wnt signaling at the start of the implantation window and suggested a prominent role for the specialized subluminal ECM in anchoring the implanting embryo (fig. S4A). Last, we examined whether embryonic fitness signals affect stromal or uNK cell dynamics during the implantation window. Random tissue fragments from midluteal endometrial samples were exposed to pooled spent medium of IVF (in vitro fertilization) blastocysts cultured in individual droplets, which subsequently implanted successfully (*n* = 40) or not (*n* = 40) (tables S2 and S3). Unconditioned blastocyst culture medium from “empty” droplets was pooled for control experiments (Fig. 3J). Notably, secreted signals emanating from unsuccessful blastocysts enhanced and inhibited *DIO2* and *PLA2G2A* expression, respectively (Fig. 3K). Inhibition of *PLA2G2A* expression was paralleled by repression of *IL15* and other decidual genes (fig. S3E). Spent medium of successful embryos, on the other hand, had no discernible impact on stromal marker genes but increased *ITGAD* expression (Fig. 3K). Together, our observations demonstrate that the balance of *DIO2*^+^ and *PLA2G2A*^+^ subsets is finely poised during the implantation window, controlled by the opposing actions of circulating progesterone levels and local T3 production. Low-fitness embryos may upend this bistable state in a manner predicted to preclude decidual transformation and promote tissue breakdown. In agreement with previous studies (*15*, *36*), expansion of *ITGAD*^+^ uNK subsets during the implantation window appears regulated by local endometrial factors, such as IL-15 and glycodelin, and likely accelerates upon implantation of a high-fitness embryo.

### The recurrence risk of miscarriage

**Fig. 4.**
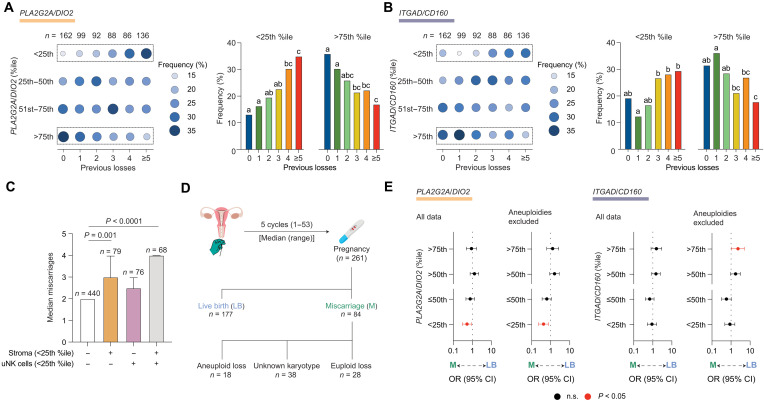
The recurrence risk of miscarriage. (**A** and **B**) Dot plots (left) showing the frequency of endometrial samples (percentage) stratified by the number of previous pregnancy losses for (A) *PLA2G2A/DIO2* percentile quartile bins and (B) *ITGAD*/*CD160* percentile quartile bins. The number of subjects in each clinical group is also shown. The frequency of samples in the lowest and highest quartile bins is also enumerated in bar graphs (right) for (A) normalized stromal subset ratios and (B) normalized uNK subset ratios. Different letters above the bars indicate significance between groups at *P* < 0.05, chi-square test with Bonferroni correction. (**C**) Median number of prior miscarriages in subjects with a stalled decidual reaction (normalized stromal subset ratios < 25th percentile), poor uNK cells expansion (normalized uNK subset ratios < 25th percentile), or both. Statistical analysis is based on one-way analysis of variance (ANOVA) and Tukey’s multiple comparisons test. (**D**) Schematic of the cohort. (**E**) Forest plots showing ORs and 95% CIs for miscarriage risk and live birth rates following assessment in a nonconception cycle of the decidual reaction (*PLA2G2A/DIO2* percentile) and uNK cell expansion (*ITGAD/CD160* percentile). Subjects were grouped in quartile bins. Data are shown for all subjects (left) and upon exclusion of confirmed aneuploid pregnancy losses (right); n.s., not significant.

We reasoned that a stalled decidual response or poor uNK cell expansion in a conception cycle increases the risk of a miscarriage. We first explored this possibility by analyzing endometrial biopsies from 663 subjects selected on the number of previous pregnancy losses (table S4), a proxy measure of age-independent miscarriage risk (*7*, *8*). The frequency of samples with a blunted or stalled decidual response (*PLA2G2A*/*DIO2 <* 25th percentile) increased stepwise with each prior miscarriage, mirrored by a stepwise decrease in samples with a heightened decidual reaction (*PLA2G2A*/*DIO2* > 75th percentile) (Fig. 4A and fig. S5). The frequency of samples with suboptimal uNK cell expansion (*ITGAD*/*CD160* < 25th percentile) increased after three or more losses (Fig. 4B and fig. S5). Women with a stalled decidual response, alone or combined with suboptimal uNK cell expansion, had more prior pregnancy losses (Fig. 4C). There was no association between the number of previous miscarriages and circulating estradiol or progesterone levels (table S5). However, circulating TSH levels at the higher end of the standard clinical range and increased body mass index (BMI) were associated with higher-order miscarriages (table S5). Increased BMI was further associated with lower stromal and uNK cell marker gene ratios (table S6). Next, we analyzed an independent cohort of 261 samples obtained before pregnancy (tables S7 and S8). The miscarriage rate in this prospective cohort was 32%, with fetal karyotyping results available for 46 of 84 miscarriages (Fig. 4D). A stalled prepregnancy decidual reaction (*PLA2G2A/DIO2* < 25th percentile) was associated with increased miscarriage risk and decreased likelihood of a live birth in a future conception cycle [odd ratio (OR), 0.52; 95% confidence interval (CI), 0.29 to 0.92, *P* = 0.02] (Fig. 4E, left). This association was more robust upon omission of confirmed aneuploid losses from the outcome data (OR, 0.42; 95% CI, 0.23 to 0.77, *P* = 0.005). Exclusion of aneuploid miscarriages also revealed that *ITGAD*/*CD160* ratios > 75th percentile favor live births in a subsequent pregnancy (OR, 2.29; 95% CI, 1.05 to 4.99, *P* = 0.04) (Fig. 4E, right).

**Fig. 5.**
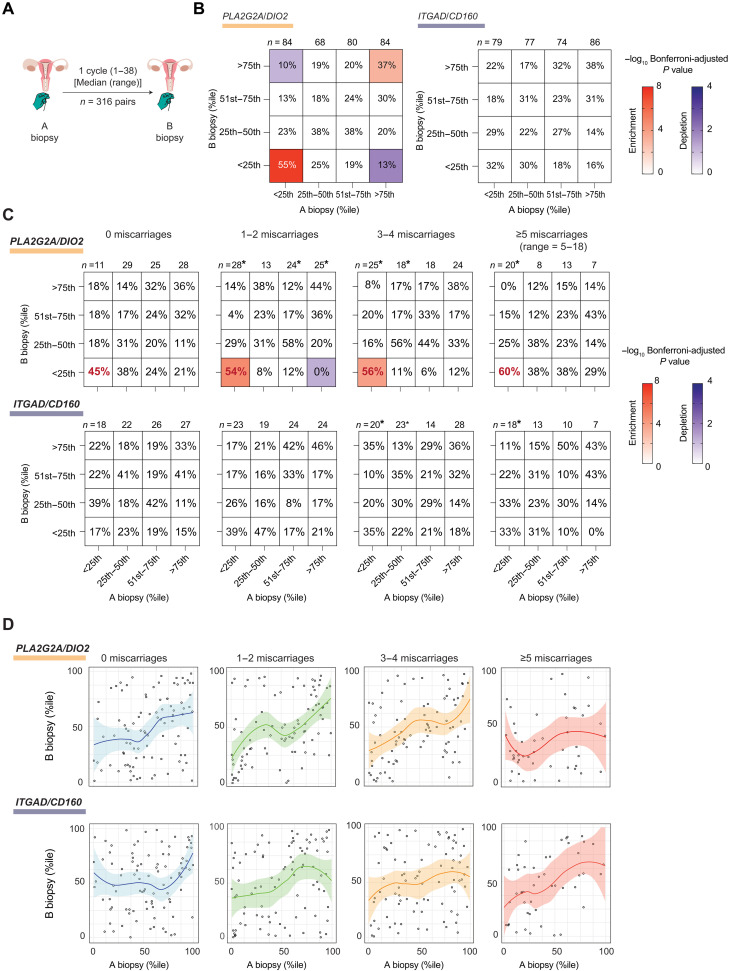
Analysis of intercycle variability. (**A**) Schematic of paired endometrial sample collection. (**B**) Contingency tables showing the distribution (percentage), grouped in quartile bins, of normalized stromal and uNK subset ratios (*PLA2G2A/DIO2* percentile and *ITGAD/CD160* percentile, respectively) in B samples for each A sample quartile bin. The number (*n*) of paired biopsies in each A sample quartile bin is shown. The colored squares in the contingency tables indicate statistical significance (*P* < 0.05), as determined by Fisher’s exact test for enriched (red key) and depleted (blue key) associations. (**C**) Contingency tables, stratified by the number of prior losses, of normalized stromal and uNK subset ratios (percentiles) grouped in quartile bins in paired A and B samples. The color key represents −log_10_ Bonferroni-adjusted *P* value, as determined by Fisher’s exact test for significantly enriched (red) and depleted (blue) associations. The recurrence rate (percentage) of a stalled decidual response is indicated in red letters. Asterisk (*) above a column in the contingency tables indicates nonuniform distribution of the relative frequency of samples in B biopsy quartile bins per A biopsy quartile bin at *P* < 0.05, chi-square test. (**D**) Scatterplots with curves of best fit (solid line) and 95% CI (colored areas).

Our findings imply that cycles characterized by an aberrant endometrial state associated with miscarriage must occur at a higher frequency than expected by chance alone. Analysis of paired endometrial samples obtained in different cycles from 316 women (Fig. 5A, fig. S6A, and table S9) showed that stalling of the decidual reaction (*PLA2G2A*/*DIO2* < 25th percentile) is significantly overrepresented in paired biopsies (Bonferroni-adjusted *P* < 1.0 × 10^−8^), with a recurrence rate of 55% (Fig. 5B and fig. S6B). An accelerated decidual response (*PLA2G2A*/*DIO2* > 75th percentile) was also overrepresented (Bonferroni-adjusted *P* < 1.9 × 10^−3^), with a recurrence rate of 36%. By contrast, the level of uNK cell expansion in the first biopsy had no discernible impact on uNK subset dynamics in the second sample (Fig. 5B and fig. S6B). The results were comparable for paired samples obtained in consecutive or nonconsecutive cycles (fig. S6C). Stratification of the data based on recurrence risk showed that one or two prior miscarriages suffice to disrupt the intercycle dynamics of stromal but not uNK subsets (Fig. 5, C and D). Further, the frequency of a stalled decidual reaction appears to increase in line with the number of prior pregnancy losses (Fig. 5C). Together, these observations highlight that the decidual reaction is intrinsically dynamic, with measurable levels of variation between cycles. Our findings further suggest that prior miscarriages compromise endometrial homeostasis, defined as the ability to maintain the decidual reaction in an optimal range from cycle to cycle.

### Impacts of prolonged decidual inflammation

**Fig. 6.**
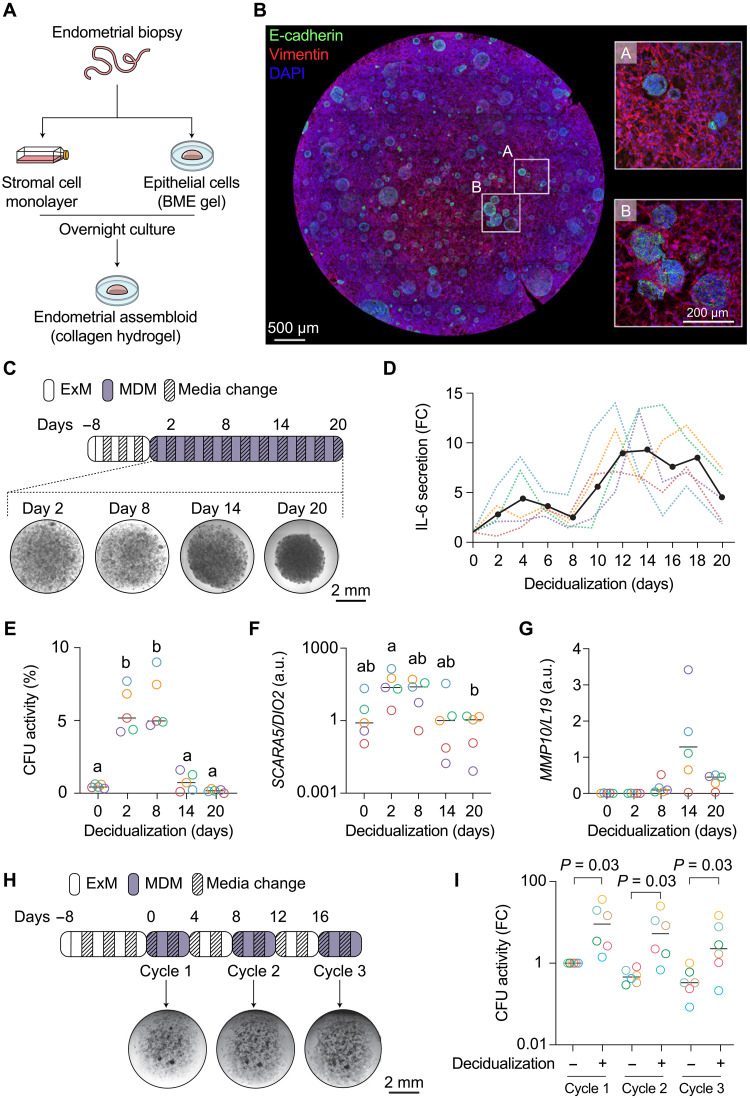
Modeling decidual inflammation in assembloids. (**A**) Schematic workflow for establishing assembloids from primary endometrial stromal and epithelial cells. BME, basement membrane extract. (**B**) Representative confocal image of E-cadherin and vimentin immunofluorescence in an assembloid grown for 8 days in a chemically defined ExM. Nuclei were stained with DAPI. Boxes highlight regions at higher magnification. (**C**) Schematic of experimental design and representative micrographs of assembloids maintained in an MDM. (**D**) FC in IL-6 secretion upon decidualization of endometrial assembloids. Colored dotted lines represent the FC in secreted IL-6 levels in five biological repeat experiments. The black solid line represents the median FC. (**E**) CFU activity in undifferentiated and decidualized endometrial assembloids. The data show median CFU activity (percentage) and individual data points of five biological repeat experiments. Different letters above the data points indicate significance at *P* < 0.05, Kruskal-Wallis test with Dunn’s multiple comparisons test. (**F**) Relative change in the *SCARA5* and *DIO2* mRNA ratio in five biological repeat experiments. Different letters above the data points indicate significance at *P* < 0.05, Kruskal-Wallis test with Dunn’s multiple comparisons test. (**G**) Time-dependent induction of *MMP10* expression. The data show median *MMP10* transcript levels, normalized to *L19*, and individual data points of five biological repeat experiments. (**H**) Schematic of experimental design and representative micrographs of assembloids first grown in ExM for 8 days and then alternatingly in MDM and ExM over 3 cycles. (**I**) Relative change in CFU activity in assembloids across 3 “cycles” of decidualization. The data show median FC in CFU activity and individual data points of five biological repeat experiments. Statistical analysis is based on Wilcoxon matched-pairs signed-rank test for comparison within each cycle.

In multiple physiological processes, including tissue repair, the transient release of proinflammatory mediators such as IL-6 enhances cellular plasticity by promoting the dedifferentiation of specified cells into stem cells (*37*, *38*). However, chronic sterile inflammation causes stem cell depletion and tissue dysfunction (*39*). Miscarriage is linked to senescence-associated inflammation, culminating in the destruction of the nascent uteroplacental interface (*40*). To explore how a miscarriage could affect stromal cell responses in subsequent cycles, we modeled the sequelae of prolonged decidual inflammation in assembloids (table S2). Following a short preassembly culture step, primary stromal and epithelial cells were mixed in a collagen hydrogel and grown into assembloids using a chemically defined expansion medium (ExM) (Fig. 6A and table S10) (*21*). After 8 days of growth (Fig. 6B), the cultures were differentiated for 20 days in minimal decidualization medium (MDM) (Fig. 6C and table S10). The medium was refreshed every 48 hours. Secretion of prolactin and insulin-like growth factor binding protein 1, markers of decidual and decidual-like senescent cells (*19*), increased across the time course (fig. S7A). By contrast, IL-6 levels followed a triphasic pattern with a transient peak around day 4 of decidualization and conspicuous secondary rise from day 10 to 12 onward (Fig. 6D), reflecting the absence of immune surveillance of decidual-like senescent cells (*19*, *21*). The initial inflammatory decidual response coincided with marked induction in the colony-forming unit (CFU) activity, measuring the relative abundance of clonogenic stem–like cells (*18*). Sustained decidual inflammation, however, abrogated this response (Fig. 6E). We used *SCARA5*/*DIO2* mRNA ratios to monitor progesterone responsiveness, as cultured cells do not express *PLA2G2A*. Prolonged decidual inflammation resulted in progesterone resistance (Fig. 6F) and induction of *MMP10* (Fig. 6G), a matrix metalloproteinase (MMP) expressed by stromal cells at menstruation (*41*). All assembloids contracted and detached spontaneously from the culture plates after 14 days in culture (Fig. 6C). By contrast, assembloids exposed alternatingly every 4 days to growth and decidualization medium not only maintained their integrity but also each “cycle” of decidualization increased CFU activity (Fig. 6, H and I, and fig. S7B). The data suggest that the decidual reaction enhances tissue plasticity in cycling endometrium. In the context of a clinical miscarriage, however, pathological decidual inflammation sustained over weeks may cause stem cell depletion, progesterone resistance, and, plausibly, disruption of the regenerative endometrial-myometrial interface (*2*).

## DISCUSSION

The functional integrity of most adult tissues relies on homeostatic circuits that sense and respond to environmental perturbations. Cues that overwhelm the capacity of these circuits trigger sterile inflammation, which serves to restore and recalibrate homeostasis (*42*). The cyclically regenerating endometrium defies the definition of a homeostatic tissue as its core functions—implantation and formation of a stable uteroplacental interface—depend on an endogenous inflammatory reaction, triggered and constrained by progesterone (*2*). By repressing stress-activated signaling pathways, progesterone not only drives the transcriptional reprogramming of stromal cells into anti-inflammatory decidual cells, which form the placental bed in pregnancy, but also causes menstruation when progesterone levels fall in nonconception cycles (*2*, *5*). We identified *PLA2G2A*, an acute-phase inflammatory gene involved in antimicrobial defenses, tissue regeneration, and eicosanoid production (*22*, *43*), as a sensitive marker gene of progesterone-dependent predecidual cells. We also identified acutely stressed source cells as putative drivers of the decidual reaction, although their provenance is uncertain. Spatial mapping revealed that progesterone-resistant *DIO2*^+^ cells abutting the luminal epithelium constrain the decidual reaction at the start of the implantation window. *DIO2*^+^ cells express genes involved in EMT and uterine patterning during development. This gene signature implicates Wnt7a, secreted selectively by proliferative phase luminal epithelium (*44*), in imposing positional identity on subluminal stromal cells (*27*). Multiple strands of evidence indicate that *DIO2*^+^ cells are essential for interstitial implantation, including the overrepresentation of genes predicted to mediate blastocyst-endometrium interactions, the expression of specialized ECM genes, and the inferred motile phenotype. By engaging placental cells, uNK cells are evolutionarily conserved partners of decidual cells (*2*, *5*, *29*). We observed that the proliferative expansion of uNK cells leads to the crowding of immunotolerant *ITGAD*^+^ (KIR^+^) subsets in the implantation niche. By contrast, cytolytic *CD160*^+^ (KIR^−^) uNK cells, tasked with curtailing decidual inflammation by pruning acutely stressed and damaged cells (*2*, *18*), reside with underlying IL-15–producing *PLA2G2A*^+^ cells.

Our findings imply that the progressive loss of *DIO2*^+^ cells and reciprocal expansion of *PLA2G2A*^+^ cells set the spatiotemporal boundaries of the interstitial implantation niche. In line with this paradigm, an accelerated decidual response is clinically associated with recurrent IVF failure (*45*), whereas delayed implantation beyond the midluteal phase—a predicted consequence of a stalled decidual reaction—markedly increases miscarriage rates (*46*). Although the mechanisms controlling time-sensitive rebalancing of stromal subsets require further investigation, we noted that signals from low-fitness IVF blastocysts, defined by their failure to implant, inhibit the decidual reaction. By contrast, successful embryos produce signals that accelerate the expansion of immunotolerant uNK cells. Thus, by imposing a bistable state on the endometrium, the decidual reaction establishes an “implantation checkpoint” that limits the risk of prolonged maternal investment in low-fitness embryos (*2*, *3*, *6*).

We report that the age-independent recurrence risk of miscarriage aligns with the frequency of a stalled decidual reaction. We found that suboptimal expansion of immunotolerant uNK cells is associated with higher-order miscarriages, possibly reflecting the impact of comorbidities associated with uNK cell dysfunction, such as obesity (*47*). Notably, strong uNK cell expansion was associated with increased likelihood of a successful future pregnancy. The management of recurrent miscarriage, defined clinically by two or three prior pregnancy losses, focuses mainly on maternal risk factors (*1*, *48*). However, the impacts of comorbidities and lifestyle factors on miscarriage rates are modest (*49*, *50*), and evidence-based treatments are, in most instances, not available (*1*, *48*). Our findings intimate that prior miscarriages compromise the ability of the endometrium to maintain a calibrated decidual reaction in subsequent cycles, thus enhancing the likelihood of further losses irrespective of embryo ploidy or maternal age. Prolonged decidual inflammation in assembloids leads to progesterone resistance, stem cell depletion, and loss of the structural integrity of the cultures, three intertwined pathological endometrial features linked to miscarriage risk. We previously demonstrated that stem cell depletion in midluteal endometrium corresponds to the number of prior losses (*51*). Imaging studies reported that disruption of the uterine junctional zone, that is, the myometrial portion of the regenerative layer (*2*, *52*), is prevalent in recurrent miscarriage (*53*, *54*). Inhibitory cytokines emanating from tertiary lymphoid structures in the regenerative layer ensure that endometrial cells become more hormone responsive with increasing distance from the endometrial-myometrial interface (*2*). Hence, progesterone resistance, culminating in a poor decidual reaction, may partly reflect the loss of spatial patterning caused by disruption of the regenerative layer. In line with this conjecture, a recent prospective study reported that sonographic evidence of junctional zone disruption does not affect implantation rates but significantly increases miscarriage risk independently of embryo quality (*54*).

Our study has limitations. We evidenced the biological plausibility that a miscarriage increases the risk of further pregnancy losses directly by disrupting the mechanisms that govern intercycle homeostasis. These mechanisms, however, remain poorly understood, and causal evidence requires carefully designed longitudinal studies and interventional clinical trials. Further, miscarriage is an umbrella term that encompasses different clinical presentations (*1*, *55*). Stalling of the decidual reaction is anticipated to cause the breakdown of the nascent uteroplacental interface and bleeding in early gestation. Thus, the relevance of our findings to other recurrent clinical presentations, such as missed (silent) miscarriages, is unclear. Last, our study focuses on stromal and uNK cell dynamics in midluteal endometrium; the contributions of other cellular constituents, including epithelial, vascular, and myeloid populations and the endometrial microbiome (*56*), to miscarriage risk warrant further exploration.

In summary, the incidence of chromosomal errors in oocytes and embryos accounts for the rise in miscarriage rates with advancing maternal age (*10*, *11*). Here, we report that the frequency of menstrual cycles resulting in a suboptimal decidual reaction determines the age-independent recurrence risk. We posit that the likelihood of one (or both) of these independent events—implantation of an aneuploid embryo and stalling of the decidual reaction—occurring in a conception cycle explains miscarriage risk in line with epidemiological observations (*3*, *8*, *57*). This paradigm is compatible with the relatively high live birth rates following multiple pregnancy losses, especially in women aged 35 or younger, and explains why treatments initiated in pregnancy that target subclinical maternal comorbidities are generally ineffective (*1*, *48*). Our findings provide the foundations for the development of endometrial tests to identify women at risk of miscarriage and evaluate prepregnancy therapeutics. When combined with other emerging miscarriage diagnostics, such as routine assessment of fetal ploidy status using circulating cell–free fetal DNA–based testing (*58*), novel strategies that improve pregnancy outcomes for the many women and their partners affected by miscarriage are bound to emerge.

## MATERIALS AND METHODS

### Study design

The primary objective of this study was to elucidate the risk of miscarriage imparted by a suboptimal decidual reaction. To achieve this goal, we investigated the cellular dynamics across the implantation window, established methods to measure the decidual reaction and expansion of immunotolerant uNK cells, and conducted mechanistic investigations using primary cultures, ex vivo tissue samples, and assembloids. We delineated the impacts of spatial and intercycle variation, hormone levels, circulating immune cells, and embryo signals on the dynamics of stromal and uNK subsets in midluteal endometrium and assessed the decidual reaction and uNK cell expansion in defined patient cohorts.

This study was conducted at the Implantation Research Clinic, a dedicated experimental reproductive medicine clinic at University Hospitals Coventry and Warwickshire (UHCW) National Health Service (NHS) Trust, Coventry, UK. Patients attending the clinic for endometrial assessment presented with a history of one or more miscarriages and/or fertility issues, including unexplained, male factor, and tubal infertility. We defined miscarriage as the spontaneous loss of pregnancy up to 24 weeks of gestation, excluding pregnancies of unknown location, ectopic or molar pregnancies, and terminations for any reason. We adopted a broad definition of miscarriage encompassing all spontaneous pregnancy losses without a requirement for ultrasound confirmation in line with the recommendations from an international consensus development study on core outcome sets for miscarriage studies (*59*). Surplus endometrial and peripheral blood samples obtained for diagnostic purposes were processed for research. The use of surplus samples for research was approved by the Arden Tissue Bank at UHCW NHS Trust (NHS Research Ethics Committee approval: 18/WA/0356). All samples were obtained following written informed consent and in accordance with the Declaration of Helsinki (2000) guidelines. Patients in the prospective outcome cohort (table S7) were enrolled in Tommy’s Net (ISRCTN registry: ISRCTN17732518), a dedicated electronic database for miscarriage research (*60*). A total of 1555 endometrial biopsies from 1308 women were processed for this study. In addition, 322 blood samples were obtained from 301 women obtained in ovulatory cycles, defined by serum progesterone levels of >10 nM (*61*). All study participants had circulating TSH levels within the clinical reference range (0.55 to 4.78 mIU/liter). A minimal set of anonymized demographic data was collected for each endometrial sample (see tables S1 to S9). The use of spent blastocyst culture medium was approved by the Institutional Ethical Committee of the Universitair Ziekenhuis (UZ) Brussel, Belgium (BUN1432021000527). Anonymized samples were pooled on the basis of clinical outcomes (table S3). Spent medium of IVF blastocysts (*n* = 40) that resulted in negative pregnancy test 14 days following single embryo transfer was allocated to the “not pregnant” group. The “pregnant group” comprised pooled culture droplets of single embryos (*n* = 40) that resulted in ongoing pregnancies beyond 12 weeks of gestation. Data were acquired in a blinded manner but unblinded during data analysis.

### Statistical analysis and visualizations

Statistical analyses were performed using R (v4.4.0) and GraphPad Prism (v10.2). Relative expression of stromal cell (*PLA2G2A* and *DIO2*) and uNK cell (*ITGAD* and *CD160*) marker genes was determined in 791 biopsies by log-transforming droplet digital polymerase chain reaction copy numbers. The normalized expression per LH + day was fitted to a normal distribution using the “fitdistr” and “fitdist” functions from the MASS (*62*) and fitdistrplus (*63*) packages. Ratios were fitted against a gamma or a log-norm distribution depending on Cramér-von Mises criterion per LH + day. Quantiles were log-transformed. A Chi-square test of independence was used to test for significant enrichment or depletion of *PLA2G2A*/*DIO2* and *ITGAD*/*CD160* percentile bins across paired endometrial samples. For each bin, a 2-by-2 grid was tested using a one-versus-all approach. A Fisher’s exact test with Bonferroni correction was applied on stratified miscarriage data, with *P* < 0.05 considered significant. A Chi-square goodness of fit test was used to test uniformity of data in B biopsies for each A biopsy percentile bin. Curves of best fit for paired biopsies stratified by number of miscarriages were produced using ggplot2’s geom_smooth with default parameters. Multiple linear regression analysis was conducted using DATAtab (DATAtab, Graz, Austria). The model was estimated using the ordinary least squares method. The DATAtab output provided regression coefficients (β), SEs, *t* values, and *P* values. These were used to assess model significance and explanatory power, with statistical significance determined at α < 0.05. All other statistical analyses encompassed Shapiro-Wilk test for normality, followed, as appropriate, by paired *t* test, Wilcoxon signed-rank test for paired data, one-way analysis of variance (ANOVA), Kruskal-Wallis test with Dunn’s test for multiple comparisons, or one-way ANOVA or Friedman test for data with three or more groups. Visualizations were performed using ggplot2 (v3.5.1) in R or GraphPad Prism.

## Supplementary Materials

### The PDF file includes:

Materials and Methods Figs. S1 to S7 Tables S1 to S15 Legend for data files S1 and S2 References
