# Supplementary material for: Stalling of the endometrial decidual reaction determines the recurrence risk of miscarriage
Source: Sci Adv. 2025 Jun 25;11(26):eadv1988. doi: 10.1126/sciadv.adv1988 (PMC12189951; doi:10.1126/sciadv.adv1988)
Supplement: Supplementary file 1 — Materials and Methods Figs. S1 to S7 Tables S1 to S15 Legend for data files S1 and S2 References [file sciadv.adv1988_sm.pdf]

Science Advances
AAAS

Supplementary Materials for

Stalling of the endometrial decidual reaction determines the recurrence risk of miscarriage

Joanne Muter *et al.*

Corresponding author: Jan J. Brosens, j.j.brosens@warwick.ac.uk

Sci. Adv. **11**, eadv1988 (2025)
DOI: 10.1126/sciadv.adv1988

The PDF file includes:

Materials and Methods
Figs. S1 to S7
Tables S1 to S15
Legend for data files S1 and S2
References

Other Supplementary Material for this manuscript includes the following:

Data files S1 and S2

Materials and Methods

Endometrial sample collection

Anonymized endometrial samples were obtained during the luteal phase of ovulatory menstrual cycles and timed relative to the pre-ovulatory LH surge, as determined by commercially available ovulation test kits. Overt uterine pathology was excluded by vaginal ultrasonography scan prior to the biopsy. The thickness of the endometrium was measured at the maximum distance between each myometrial/endometrial interface perpendicular to the sagittal axis of the uterus. Endometrial biopsies were obtained using a Wallach Endocell™ or CerviX™ endometrial sampler. Paired endometrial biopsies were obtained in 316 women. In six cases, a miscarriage occurred between sampling of the endometrium. Two or more regular menstrual cycles were required for repeat endometrial sampling following a pregnancy loss.

Blastocyst conditioned media

Spent culture medium was collected following *in vitro* culture of human embryos created either by *in vitro* fertilization (IVF) or intracytoplasmic sperm injection (ICSI) following controlled ovarian stimulation and oocyte retrieval (*64, 65*). Human embryos were cultured for 5 days in individual 25 µL droplets of ORIGIO® Sequential Series™ medium, overlaid in OVOIL™ (Vitrolife, 10029) at 37°C, 6% CO_2, 5% O_2 in a G185 flatbed incubator (CooperSurgical, Denmark). Embryos were first cultured for 3 days in 25 µL droplets of ORIGIO® cleavage-stage medium under oil and then cultured in 25 µL droplets of ORIGIO® blastocyst medium under oil. Single day-5 embryos, selected based on morphological criteria (*66*) (Table S3), were transferred into the uterine cavity. Spent culture medium (22 µL) was collected following embryo transfer using a sterile MµltiFlex pipette tip (Sorenson BioScience, 13810), transferred to a nuclease-free cryovial (Nalgene, USA), snap frozen in liquid nitrogen and stored at -80°C.

RNA extraction and cDNA synthesis

RNA extraction from RNAlater-preserved tissues was performed using the RNeasy Plus Universal Mini Kit according to the manufacturer’s protocol (Qiagen, 73404). RNA concentrations were determined using a Nanodrop ND-1000 spectrophotometer, and equivalent RNA quantities reversed transcribed into cDNA using the QuantiTect Reverse Transcription Kit (Qiagen, 205314).

RT-qPCR

Amplification of target genes by RT-qPCR was performed on a QuantStudio 5 Real-Time PCR system (Applied Biosystems, Paisley, UK). cDNA transcribed from RNA (1 µg) was diluted to a concentration of 10 ng/µL. For the qPCR reaction, 1 µL of cDNA was combined with 9 µL of reaction mixture, which included QuantiNova SYBR Green Master Mix (Qiagen, 208057), a low concentration of ROX Dye solution, and 300 nM each of forward and reverse primers. The primer sequences are provided in Table S11.

Droplet digital polymerase chain reaction (ddPCR)

ddPCR was performed on the QX200 AutoDG Droplet Digital PCR System (Bio-Rad). Briefly, 10 ng of cDNA was added to a 19 µL reaction mixture containing 1 × supermix for probes, 900 nM target primers, and nuclease-free water. Target probes are provided in Table S12. Droplet generation was accomplished using the automated droplet generator (Bio-Rad). The droplet emulsion was subjected to thermal cycling under the following conditions: one cycle of enzyme activation (95°C, 30 sec), 40 cycles of denaturation (94°C, 30 sec), annealing/extension (55°C, 1 min) and one cycle of enzyme deactivation (98°C, 10 min). Post-cycling, PCR amplification within the droplets was assessed using the QX200 Droplet Reader (Bio-Rad). Gene expression threshold between positive and negative drops were determined using QuantaSoft software (version 2.1).

Bulk mRNA library preparation and sequencing

RNA-seq libraries were mapped to the hg38 human genome assembly using STAR v2.5.3a (*67*) using default settings and gencode.v38 as annotation file (Ensembl). Differential gene expression analysis was performed with DESeq2 v1.34.0 in R (*68*). Principle component analysis (PCA) was performed with prcomp function following transformation of raw counts using the rlog function.

Processing endometrial samples for scRNA-seq

Endometrial biopsies in additive-free DMEM/F12 media were processed within 15 min of collection. Samples were mechanically minced and enzymatically digested with DNase I (100 µg/mL), Collagenase Type Ia (500 µg/mL) and IV (10 µg/mL) for 90 min at 37°C, with shaking every 15 min. Undigested tissue was removed through a 40 µm cell strainer. To eliminate red blood cells, the cell suspension was incubated with red blood cell lysis buffer (Invitrogen, 00-4333-57) for 5 min at room temperature. The reaction was terminated by

adding 30 mL PBS and centrifuged at 400 × *g* for 5 min. Cells were then processed according to Demonstrated Protocol CG00054 (Revision B, 10X Genomics) with some modifications. Briefly, cells were centrifuged at 300 × *g* for 5 min and resuspended in 0.04% BSA in PBS. This was repeated twice more before cells were resuspended in 500 µL PBS with 0.04% BSA and counted. Cells were adjusted to 700 cells/µL.

Single-cell capture and library preparation

Freshly digested endometrial cells were encapsulated into emulsion droplets using the 10X Genomics Chromium Controller and NextGEM Single Cell 3' Reagent Kit (v3.1), according to the user guide CG000315, Rev B, targeting 8000-10000 cells per run. Libraries were constructed as per manufacturer's instructions. Quality control and library quantitation were performed using the Agilent Bioanalyzer and KAPA Library Quantification Kit (KAPA Biosystems, KK4824). Sequencing was performed on a NextSeq 500, using Illumina NextSeq500/550 High Output Kits, v2.5 (150 cycles) with read lengths as described by 10X Genomics (Read 1: 28, i7 Index: 10, i5 Index: 10, Read 2: 90), targeting 20,000 read pairs per cell.

Single-cell data pre-processing and quality control

Raw sequencing data was processed using CellRanger v6.1 pipelines `cellranger mkfastq` and `cellranger count` (10X Genomics). SoupX (*69*) was used to remove ambient RNA contamination, and count matrices were processed using Seurat v4.4 (*70*). Cells were excluded from the analysis if they had less than 200 detected genes or more than 25 % mitochondrial gene expression. DoubletFinder (*71*) (version 2.0.4) was used to predict and remove cell doublets. Integration was performed across patient samples using the reciprocal PCA method. A total of 64,644 high-quality endometrial cells were retained for further bioinformatic analysis.

Single-cell data integration, dimensionality reduction and cell annotation

Global scaling normalization was applied through the NormalizeData function. Highly variable genes were identified through FindVariableGenes function. For batch correction, reciprocal PCA was applied to data integration. PCA was performed on the integrated dataset. Top 20 PCs were selected for downstream analysis with the Elbowplot function. The main cell clusters were identified with the Seurat FindClusters function with the resolution set at 0.5. The endometrial cells were clustered and visualized by uniform manifold approximation and

projection (UMAP) plots. scCustomize package (*72*) was used to visualize gene expression. Cell type identification was performed by manual inspection of marker genes and interpretation of these based on previous studies. Subsequently, stromal cells and uNK cells were further subclustered to detect heterogeneity. The differential gene expression analysis of identified cell clusters was executed using the Wilcoxon rank-sum test within the Seurat's FindAllMarkers command. We used the GSEA (*73*) with hallmark gene sets to identify biological pathways enriched in endometrial stromal subsets (Data file S1).

Receptor-ligand interactions

CellPhoneDB v4 (*74*) with default parameters was used to predict enriched receptor-ligand interactions involved in the crosstalk between human trophectoderm from day-6/7 blastocysts and endometrial subsets. Endometrial subpopulations were identified based on thresholding (>0) of marker gene counts. scRNA-seq data from human embryos (E-MTAB-3929) (*75*) were normalized using Seurat's SCTransform and then clustered using FindClusters at resolution 0.5. Clusters were identified as trophectoderm, epiblast or hypoblast through VlnPlot using relevant biomarkers. The datasets were merged, and counts were globally normalized using SCTransform. Default parameters with temporally separated endometrial microenvironments (early-, mid-, late-secretory phase) were used for CellPhoneDB. For each microenvironment, all embryonic cells were included. Annotated temporal interactions were curated manually. The receptor-ligand interactions are listed in Data file S1.

Single-cell RNA velocity

RNA velocity analysis, which calculates the ratio of unspliced to spliced RNA for each gene, was performed to identify the direction of differentiation (*76, 77*). Fastq files were first processed using loompy Python package v3.0.7 (fromfq using gencode. v31.600. Kallisto v0.46.2). The RNA velocity estimation was conducted using scVelo package v0.2.4 in Python v3.9.13 (*77*) , with a specific focus on visualizing RNA velocity through stream plots. The data were pre-processed to filter low-quality cells and genes, normalize the data, and compute moments essential for accurate velocity modelling with functions `scv.pp.filter_and_normalize` and `scv.pp.moments`. The stochastic model was employed for velocity estimation using `scv.tl.velocity`, which accounts for probabilistic transcriptional dynamics. A key aspect of our analysis was the integration of precomputed UMAP embeddings from a previous analysis, allowing visualization of RNA velocity in the biologically relevant UMAP space. To visualize the RNA velocity, we used stream plots,

generated by the function `scv.pl.velocity_embedding_stream`. This approach overlays velocity vectors in the form of arrows, which represent the direction and magnitude of RNA velocity on the UMAP. This visualization highlights the continuous flow of cellular transitions, providing an intuitive representation of differentiation trajectories.

Single-cell analysis of transposable element (TE)-derived transcripts

Locus-specific quantification of TE-derived transcripts in the transcriptomic data was performed with SoloTE (*78*). The resulting count matrix was used for single-cell analysis using Seurat.

Multiplexed single molecule in situ hybridization (smISH)

Endometrial biopsies were fixed overnight in 10% neutral buffered formalin. Tissue processing through graded alcohol and embedding in Surgipath Formula 'R' paraffin was automated using the Shandon Excelsior ES Tissue Processor, and a Tissue-Tek TEC embedder. Tissues were sectioned at 5 μM and mounted on SuperFrost Plus slides (Epredia, J1800AMNZ). Staining was carried out using the RNAscope Multiplex Fluorescent Reagent Kit v2 Assay (Advanced Cell Diagnostics, Bio-Techne). The probes are listed in Table S13.

Visium spatial transcriptomics

Formalin-fixed, paraffin-embedding human endometrial tissue sections (5um thick) were mounted onto Visium Spatial Gene Expression slides(10x Genomics) and adhered according to the manufacturer's guidelines. Permeabilization conditions were optimized using a permeabilization time of 30 minutes as determined by the Tissue Optimization kit (PN-1000194). Following permeabilization, reverse transcription was performed using the Visium Spatial Gene Expression Kit (10 x Genomics, PN-1000187), generating spatially barcoded cDNA. cDNA libraries were constructed following the 10x Genomics Visium protocol and quantified using the Bioanalyzer and pooled at equimolar concentrations for sequencing. Sequencing was performed on an Illumina NextSeq with a tagerted depth of 25,000 reads pairs/spot, using the following parameters: 28cy R1, 10cy i7 index, 10cy i5 index, 50cy R2. Spaceranger software (version 1.3.0, 10x Genomics) was used to align and obtain raw counts from each of the spots on the Visium spatial transcriptomics slides against the GRCh38 human genome reference data (refdata-gex-GRCh38-2020-A). The spatial transcriptomics raw gene expression matrix, together with spatial location of spots and tissue hematoxylin and eosin

(H&E) images, were used to create a Seurat object with a Load10X_spatial function. After normalization by SCTransform, we performed principal component analysis and reduced the dimensions to the top 20 principal components. Marker gene detection and differential gene expression were carried out using the FindAllMarkers function in Seurat. Genes that varied by location were identified using the FindSpatiallyVariableFeatures function using default settings. The DNA repair gene signature was visualized by summing the count data of the 150 genes in the GSEA HALLMARK_DNA_REPAIR set (*73*).

Serum hormone levels

Blood was collected immediately prior to biopsy collection in serum-separating tubes. Quantitation of hormonal levels in serum was based on acridinium ester chemiluminescent technology using the Atellica IM Analyser system (Siemens Healthcare GmbH, Erlangen, Germany). TSH (Atellica IM TSH3 UL) was quantified through direct analysis utilizing FITC-labelled anti-TSH capture mouse monoclonal antibody, this was bound to the solid phase via an anti-FITC monoclonal antibody. Introduction of an anti-mouse monoclonal antibody labelled with acridinium initiated the reaction and a direct relationship was observed between the amount of TSH in the sample and relative light units detected. Progesterone (Atellica IM PRGE assay) and estradiol (Atellica IM E2 assay) were measured using a competitive immunoassay format. The target analyte was first bound to an acridinium-ester-labelled monoclonal anti-hormone antibody. A derivative of the target hormone coupled to the capture solid phase was then introduced, which competes and binds any remaining antibody. Following a wash, the addition of acid and base to initiate the reaction resulted in an inverse relationship between the amount of hormone in the sample and relative light units detected. Samples were processed in a fully UKAS accredited medical diagnostic laboratory working to the international ISO 15189:2012 standard.

Immunohistochemistry

Formalin-fixed, paraffin-embedded human endometrial tissue sections (3 μm thick) were deparaffinized three times with xylene, 5 min each, and rehydrated using 100% isopropanol (2×) and 70% isopropanol (1×), each for 5 min. Antigen retrieval was performed in citrate buffer (10mM citrate, 0.05% Tween 20, pH 6). After blocking with 2% BSA in TBST, sections were incubated with indicated primary antibodies overnight at 4°C. Subsequent washing with TBST was followed by incubation with Alexa-fluor 594 anti-mouse or Alexa-fluor 488 anti-

rabbit secondary antibodies for 2 hours at room temperature. After three TBST washes, the sections were treated with Vector TrueVIEW auto-fluorescent quenching solution (Vector Laboratories, SP-8400). The slides were washed with PBS and cover slipped using the ProLong™ Gold Antifade Mountant with DAPI DNA Stain. Imaging was conducted using a BioTek Cytation C10 confocal imaging reader (Agilent Technologies) and analyzed using Gen5 image analysis software version 3.12. ImageJ/Fiji software was used for editing purposes (*79, 80*). The antibodies are listed in Table S14.

Flow cytometry and sorting of primary uNK cells

Following tissue digestion, endometrial single-cell suspension was incubated with red blood cell lysis buffer (Invitrogen, 00-4333-57) for 5 min at room temperature. The reaction was terminated by adding 30 mL PBS and centrifuged at 400 × *g* for 5 min. The single-cell suspension was washed twice with PBS (300 × *g* for 5 min) and incubated with fluorescent-conjugated antibodies in a wash buffer (0.5% BSA in PBS with 2.5 mM EDTA, 4°C). Live and dead cells were discriminated using Fixable Viability Stain 660 (1:1000) in PBS. Cell phenotyping and sorting were conducted on a Becton Dickinson (BD) FACS Melody with 3 lasers (488 nm, 561 nm, and 640 nm) and 8 detectors. Sorting utilized BD FACS Chorus software (version 3.0). Analyses were performed using FlowJo software (version 10.9.0). Antibodies are listed in Table S15.

Peripheral blood lymphocyte immunophenotyping

Whole blood drawn into BD EDTA Vacutainers and BD Vacutainer® CPT™ Mononuclear Cell Preparation Tubes with Sodium Heparin (BD Biosciences, 362753) was used for T, B, and NK cell (TBNK) assays (BD Biosciences, 644611). A lyse/no-wash method was employed by adding 20 µL of BD Multitest™ 6-color TBNK Reagent to BD Trucount™ tubes (BD Biosciences) and mixing it with 50 µL of anticoagulated whole blood. The tubes were gently vortexed and incubated at room temperature for 15 min. Subsequently, 450 µL of Lysing solution (BD Biosciences) was added, followed by another 15 min incubation at room temperature. The samples were then analyzed using the BD FACSCanto™ II cytometer, and population numbers were calculated with BD FACSDiva™ Software (version 9.2). Leukocytes were gated based on CD45 positivity and side scatter. Within this gate, the relative abundance of CD56+/CD16+ NK cells and CD3+ T cells was quantified.

Endometrial explant culture

Fresh endometrial tissue samples divided into pieces within 1-2 min of sample collection were added to 1.5 ml Eppendorf tubes containing additive-free DMEM/F12 medium with or without T3 (50 pM) or blastocyst conditioned medium (1:1 dilution with additive-free media). Samples were maintained at 37 °C in 5% CO_2 on a rocking platform with a frequency of 0.1 Hz. After 3 hours, tissues were immersed in RNAlater for RNA extraction, cDNA synthesis, and ddPCR analysis.

Isolation of primary stromal and epithelial cells

Endometrial biopsies were chopped using scalpels for 5 min and enzymatically digested in 5 mL of pre-warmed additive-free DMEM/F12 medium containing 500 μg/mL Collagenase Type Ia (Merck Life Science, C9891) and 100 μg/mL DNase I (Lorne Laboratories LTD, LS002060) for 1 hour at 37 °C. Cell suspension was then filtered through 40 μm cell strainer to separate epithelial cells. The flowthrough contained stromal cells, and the strainers were inverted and back-washed to collect epithelial cells. Cells were centrifuged (300 × *g*) for 5 min and then resuspend in 10% DMEM/F12 growth medium (composition: DMEM/F12 with phenol red supplemented with 10% dextran-coated charcoal-stripped foetal bovine serum (DCC-FBS), Antibiotic-Antimycotic mix (Gibco, 15240062), 2 mM L-glutamine (Gibco, 25030081), 1 nM β-estradiol (Merck Life Science, E2758) and 2 μg/mL recombinant human insulin (Merck Life Science, 91077C). Cells were routinely cultured at 37 ºC in a 5% CO_2, humidified environment.

Decidualization of primary stromal cells and uNK cell co-cultures

Non-passaged endometrial stromal cells grown to confluency were then incubated overnight in 2% DMEM/F12 reduced-serum media (composition: DMEM/F12 medium without phenol red supplemented with 2% DCC-FBS, Antibiotic-Antimycotic mix and 2 mM L-glutamine) to synchronize the cell cycle. For decidualization, cells were treated with 0.5 mM 8-bromo-cAMP (Merck Life Science, B7880) and 1 μM medroxyprogesterone acetate (MPA) (Merck Life Science, M1629) in 2% DMEM/F12. For stromal-uNK cell co-cultures, $KIR^{+/-}$ uNK cell subsets were isolated by FACS and added (50,000 events/well) directly to primary stromal cell cultures first decidualized for 6 days in 96-well plates. The co-cultures further treated with 0.5 mM 8-bromo-cAMP and 1 μM MPA for two additional days. Protein lysates were then harvested for quantitation of SA-β-Gal activity.

SA-β-Gal activity quantitation

SA-β-Gal activity was measured using the 96-well Quantitative Cellular Senescence Assay kit (Cell Biolabs Inc, San Diego, U.S.A). Endometrial stromal cells were rinsed with ice-cold PBS and lysed in 50 μL of ice-cold lysis buffer containing protease inhibitors (cOmplete Protease Inhibitor Cocktail, Roche). Subsequently, 25 μL of 2 × assay buffer was added to 25 μL of cell lysates transferred into black-walled, black-bottomed 96-well plates. The plates were sealed and incubated for 1 hour at 37°C. The reaction was terminated by the addition of 200 μL stop solution. Fluorescent intensity, measured by arbitrary fluorescent intensity units (F.I.U), was determined using a PHERAstar FS plate reader (BMG Labtech) at 360/465 nm.

Endometrial assembloids

Assembloids were established as described previously (*21*), with some important modifications. Following the isolation of primary stromal and epithelial cells from endometrial biopsies, stromal cells were cultured as described above. Freshly isolated endometrial gland fragments were resuspended in 500 μL phenol red-free DMEM/F12 medium in a microcentrifuge tube and centrifuged at 600 × *g* for 5 min. The medium was aspirated and ice-cold, growth factor-reduced Cultrex RGF Basement Membrane Extract, Type 2 (BIO-TECHNE) was added at a ratio of 1:20 (cell pellet: Cultrex). Samples were aliquoted in 20 μL droplets to a 48-well plate, one drop per well, and allowed to cure for 30 min and then overlaid with 200 μL of expansion medium (ExM). Stromal cells and gland organoids were digested, as described previously(*21*), into single cell suspension 24 hours after establishment and mixed at a ratio of 1:1 (v/v) and ice-cold PureCol EZ Gel (Sigma-Aldrich) added at a ratio of 1:20 (cell pellet: hydrogel). The suspension was aliquoted in 20 μL droplets into a 48-well plate (1 droplet per well). Following hydrogel polymerization at 37 °C and 5% CO_2 for 60 min, droplets were overlaid with 200 μL of ExM with 10 nM β-estradiol. Medium was refreshed every 48 hours for 8 days. To induce decidualization, ExM was replaced with minimal decidualization medium (MDM) containing 1 nM β-estradiol, 0.5 mM 8-bromo-cAMP and 1 μM MPA. The composition of ExM and MDM is tabulated in Table S10.

Imaging of endometrial assembloids

For immunofluorescent microscopy, assembloids were fixed in 4% paraformaldehyde in PBS for 15 min at room temperature to crosslink proteins and preserve cellular integrity. The procedure was carried out in 1.5 mL Eppendorf tubes. After fixation, assembloids were washed

3 times with PBS-0.05% Tween 20 for 5 min and permeabilized in PBS-0.1% TritonX for 1 hour at room temperature. Assembloids were incubated in primary antibody (Table S14) diluted in antibody/blocking buffer (10%FBS, 2%BSA, 0.05%Tween 20 in PBS) overnight at 4 °C. Assembloids were then washed in PBS-0.05% Tween 20 for 3 × 5 min before incubation with the secondary antibody for 1 hour in the dark at room temperature. Following washing with PBS-0.05% Tween 20 (3 × 5 min), assembloids were incubated in Hoechst 33342 for 20 min, treated with autofluorescence quenching solution and imaged on ibidi µ-slide 8-well chamber slides (ibidi GmbH, 80826). Samples were kept in clearing solution for 3-5 days. Immunolabelled assembloids were imaged using a confocal microscope (LSM800 equipped with Airyscan, ZEISS) with z-stack scans to reconstruct the 3D structure. Z-stacks were processed with Zen-Blue software (ZEISS). The EVOS M7000 Imaging System (Thermo Fisher Scientific) was used for brightfield and phase contrast microscopy. ImageJ/Fiji software was used for editing purposes (*79, 80*).

Colony forming unit (CFU) assay

For stromal cell isolation, assembloids were washed with PBS before cell recovery by scraping. Samples transferred into microcentrifuge tubes were centrifuged at 600 × g for 6 min, resuspended in 500 µL of 500 µg/mL Collagenase I and 100 µg/mL DNase I diluted in additive-free DMEM/F12, and incubated at 37°C for 10 min with regular manual shaking to release the cells from the hydrogel. Samples were then centrifuged (600 × g, 6 min, room temperature), the pellets resuspended in 5 mL phenol red-free DMEM/F12 growth medium, and the mixtures passed through a 40 µM cell strainer to isolate stromal cells. Stromal cells were pelleted by centrifugation at 300 × *g* for 5 min and resuspended in 1 mL growth medium. Viable isolated stromal cells were counted in trypan blue on a Neubauer Improved hemocytometer, seeded at clonal density (104 cells/cm^2) onto 6-well plates coated with fibronectin (2 µg/cm^2 in PBS; Merck Life Sciences, F0895), and maintained in growth medium supplemented with 10 ng/µL basic fibroblast growth factor (PeproTech, 100-18B). Plates were cultured in 5% CO_2 at 37 °C and the media partially refreshed after 7 days. After 10 days, the cultures were washed in PBS, fixed in 10% neutral buffered formalin for 10 min at room temperature, washed again with sterile water, and stained with Harris hematoxylin for 5 min. Cells were imaged using an EVOS M7000 and analyzed in ImageJ using the cell counter plugin to enumerate clonal colonies comprising 50 cells or more.

Enzyme-linked immunosorbent assay (ELISA)

Spent media of monolayer or assembloid cultures was collected and cleared of debris by centrifugation (16000 ×*g*, 10 min at 4°C). Quantitation of analytes in cell supernatant was performed using DuoSet sandwich ELISA kits exactly (Bio-Techne, Abingdon, UK). Standard curves were fitted to a 4-parameter logistic fit curve and analyte concentrations interpolated from these graphs.

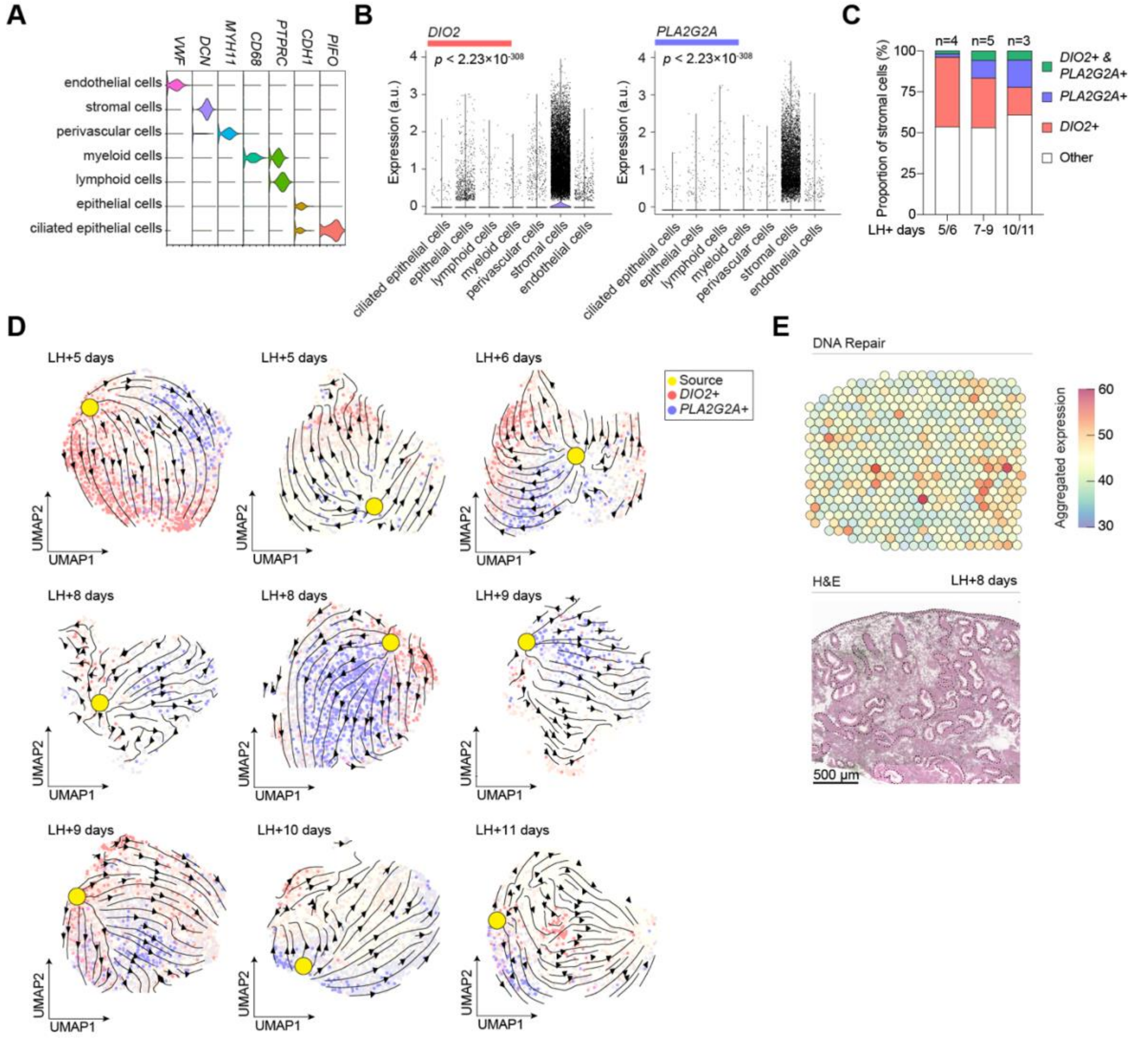

fig. S1. Single-cell transcriptomics of endometrial stromal subsets. (**A**) Violin plots showing expression of canonical marker genes in different endometrial cell types. (**B**) Violin plots showing *PLA2G2A* and *DIO2* expression across different endometrial cell types with *p*-values based on Wilcoxon rank sum test; a.u., arbitrary units. (**C**) Column bar graphs depicting the relative abundance of stromal subsets across the indicated cycle days. (**D**) RNA velocity mapped onto UMAP plots of stromal subsets in nine endometrial biopsies. (**E**) Visualization of the GSEA hallmark 'DNA Repair' gene set in mid-luteal endometrium by Visium spatial transcriptomics (upper panel). The lower panel shows the corresponding tissue section stained with H&E.

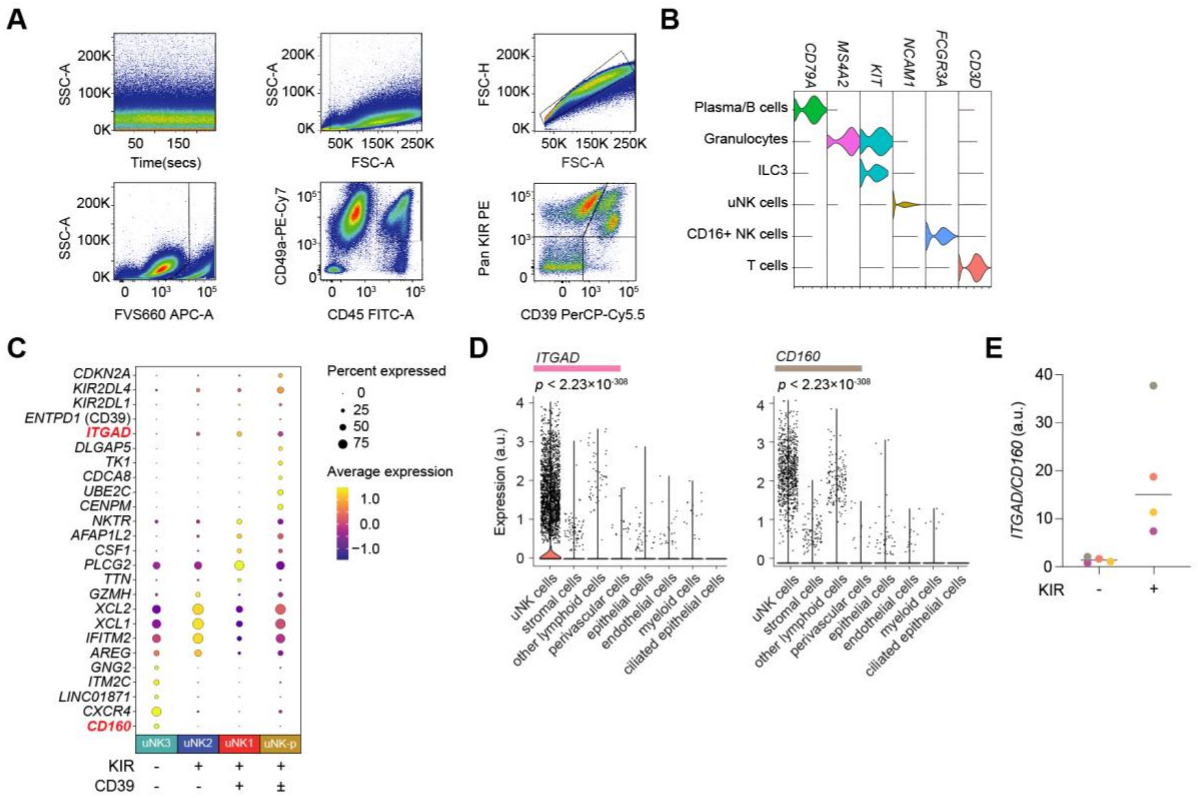

fig. S2. Analysis of uNK subsets. (**A**) Flow cytometry gating strategy for uNK cell subpopulations. (**B**) Violin plot based on scRNA-seq data of selected marker genes for different immune cell populations in peri-implantation endometrium. (**C**) Dot plot of expression levels of selected differentially expressed genes in uNK1-3 subsets; uNK-p denotes proliferating uNK cells. Dot size represents the percentage of positive cells, and the colour key indicates average gene expression levels. (**D**) Violin plots of *ITGAD* and *CD160* expression in different endometrial cell types. Statistical significance was determined using the Wilcoxon rank sum test. (**E**) Ratio of *ITGAD* and *CD160* transcripts in KIR+ and KIR- uNK subsets purified by FACS. *ITGAD* and *CD160* transcript levels were quantified by ddPCR. The data show individual ratios and median ratio from four biological repeat experiments; a.u., arbitrary units.

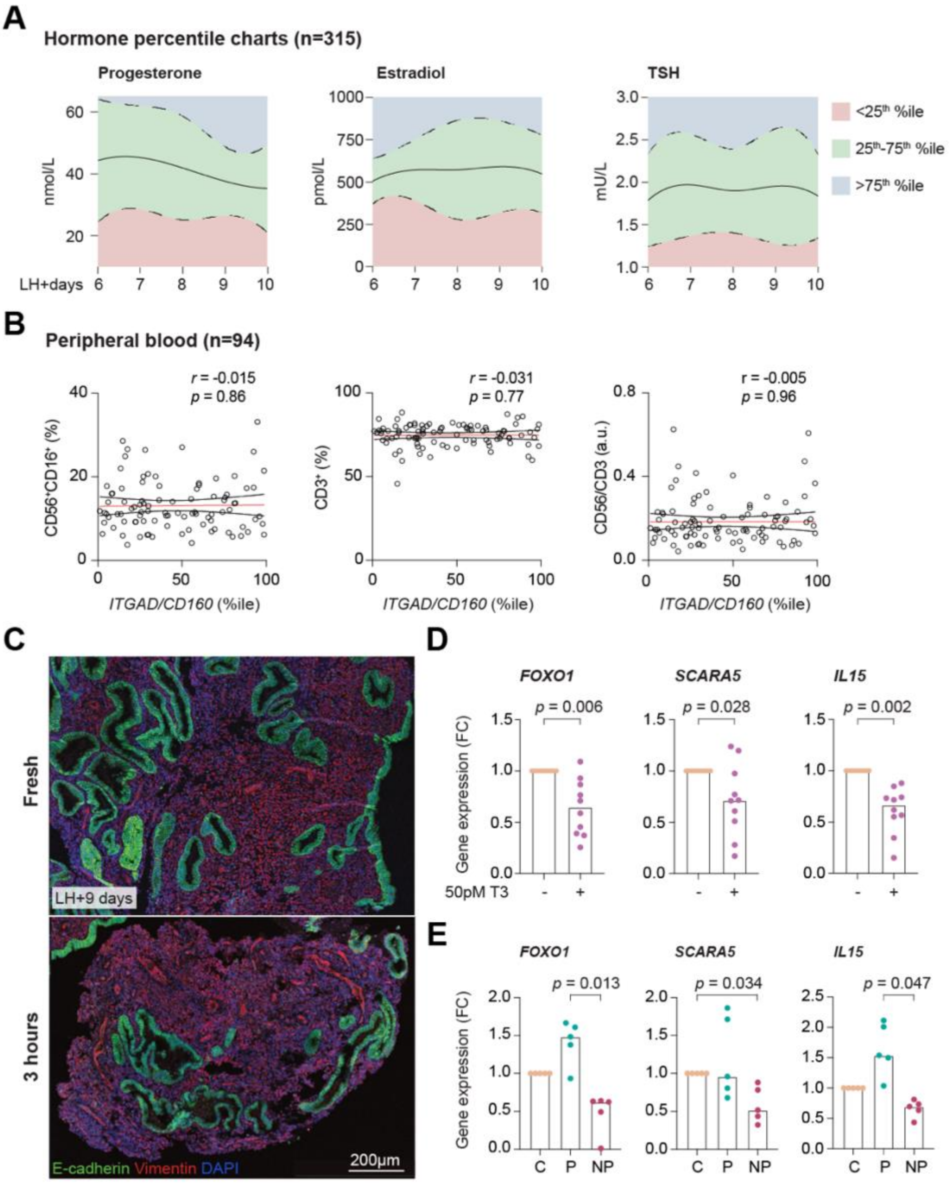

fig. S3. Hormones and peripheral blood immune cells (**A**) Circulating levels of progesterone (nmol/L), estradiol (pmol/L) and TSH (mU/L) in 315 subjects 6 to 10 days after the LH surge. The solid line denotes median circulating levels, and the dotted line show upper and lower quartiles. (**B**) Spearman's correlation between uNK cell expansion, measured by the percentile rank of normalized *ITGAD*/*CD160* ratios, and the relative abundance of circulating CD56+ NK cells, CD3+ T cells and CD56+/CD3+ cell ratios in paired peripheral blood and endometrial samples from 94 subjects. (**C**) Representative images of vimentin (red) and cadherin 1 (green) immunofluorescence in a freshly isolated tissue section before and after incubation in additive-free media (AFM). Nuclei were stained with DAPI. (**D**) Freshly isolated endometrial samples were divided, and sections incubated for 3 hours in AFM or media supplemented with 50 pM triiodothyronine (T3) prior to gene expression analysis by RT-qPCR. Bar graphs show fold change (FC) in the transcript levels of pre-decidual marker genes in response to T3 treatment in 10 biological repeat experiments. Individual data points are also shown. Statistical significance was determined using the Wilcoxon matched pairs signed rank

test. (**E**) Freshly isolated endometrial samples were divided, and sections incubated for 3 hours in pooled spent medium of IVF blastocysts, resulting in pregnancy (P) or not (NP, not pregnant). Embryo-free droplets collected in parallel served as the control (c) group. Bar graphs showing median FC in the transcript levels of pre-decidual marker genes in five biological repeat experiments. Individual data points are also shown; *p*-values based on the Friedman and Dunn's multiple comparison test.

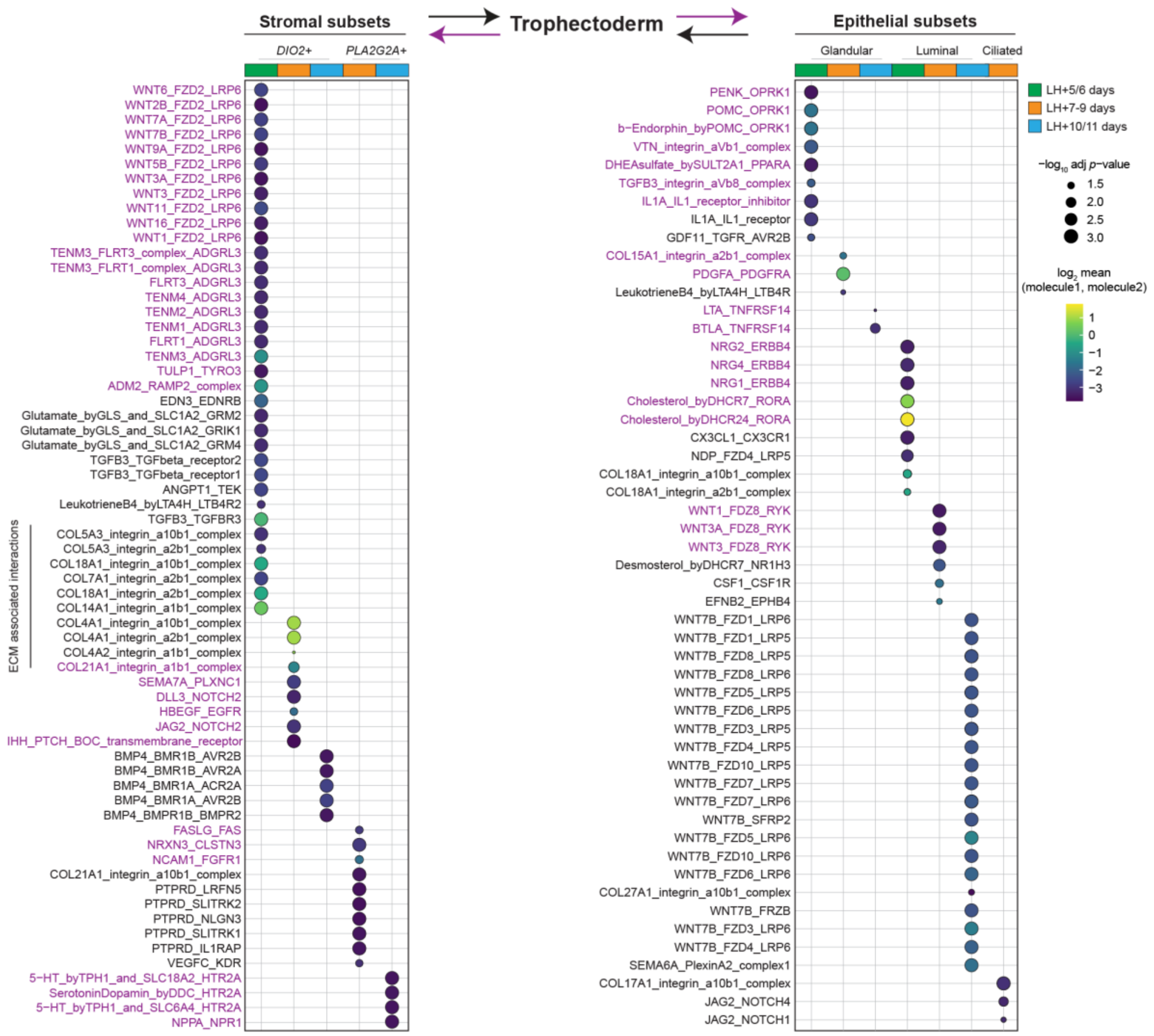

fig. S4. Embryonic trophectoderm-endometrium crosstalk. Dot plots showing CellPhoneDB predictions of receptor-ligand interactions of between endometrial stromal subsets (left panel) and epithelial cell types (right panel) cells and day-6/7 embryonic trophectoderm. Interaction involving embryonic trophectoderm and endometrial ligands are highlighted in purple and black lettering, respectively. Circle size and color scale indicate FDR-adjusted *p*-value and the mean of the average expression values of genes encoding interacting molecules, respectively.

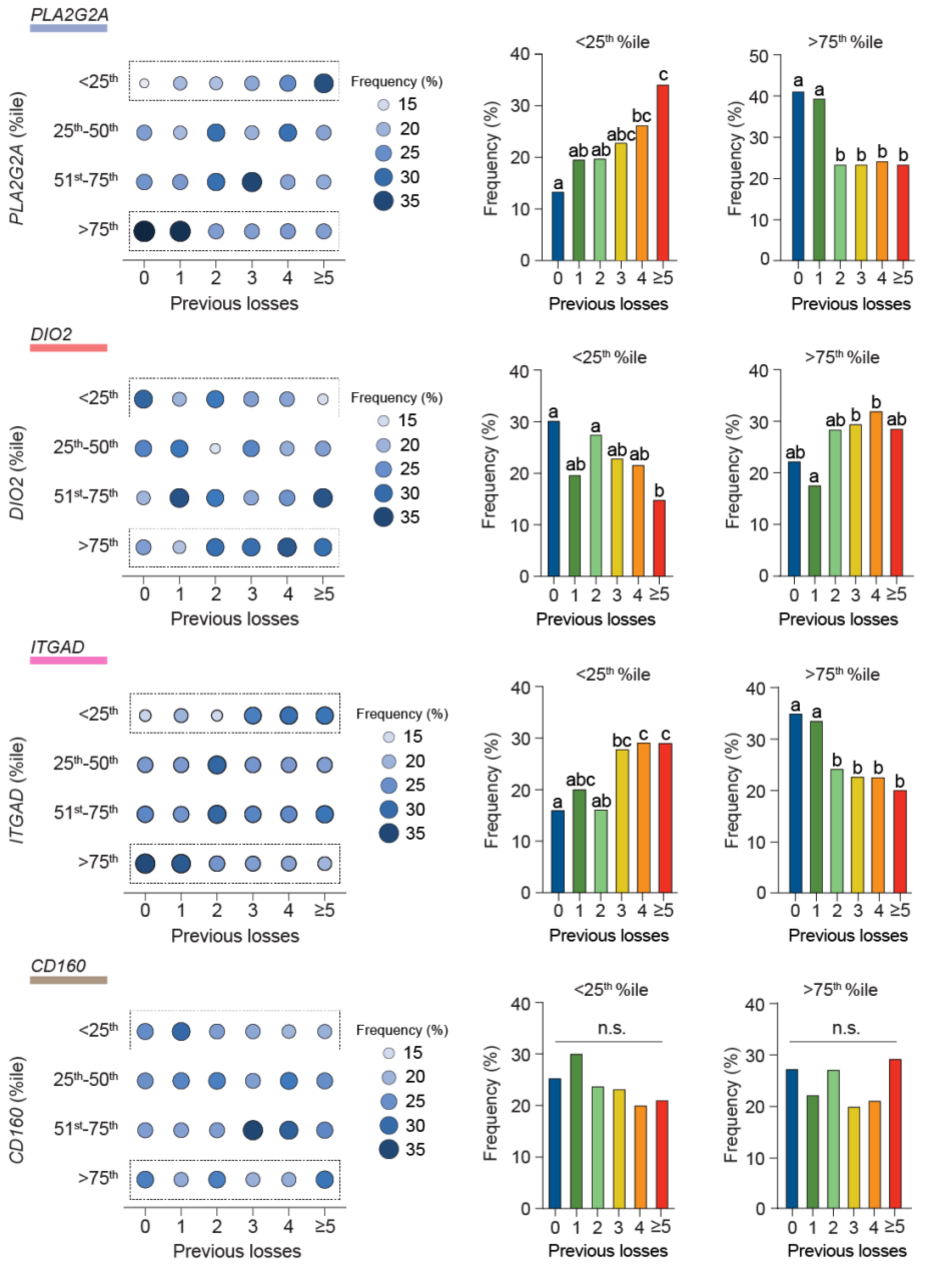

fig. S5. Stromal and uNK subset marker genes stratified by previous miscarriages. Dot plots of normalized marker genes grouped in quartile bins and stratified by the number of previous pregnancy losses (left panels). Dot sizes and color gradients denote the proportion (%) of subjects in each quartile. The number of subjects in each miscarriage group is also indicated. Bar charts (right panels) enumerating the proportion of subjects in the lowest (<25th %ile) and highest (>75th %ile) quartile bin. Different letters above the bars indicate groups that are significantly different from each other at $p < 0.05$, Chi-squared test with Bonferroni correction.

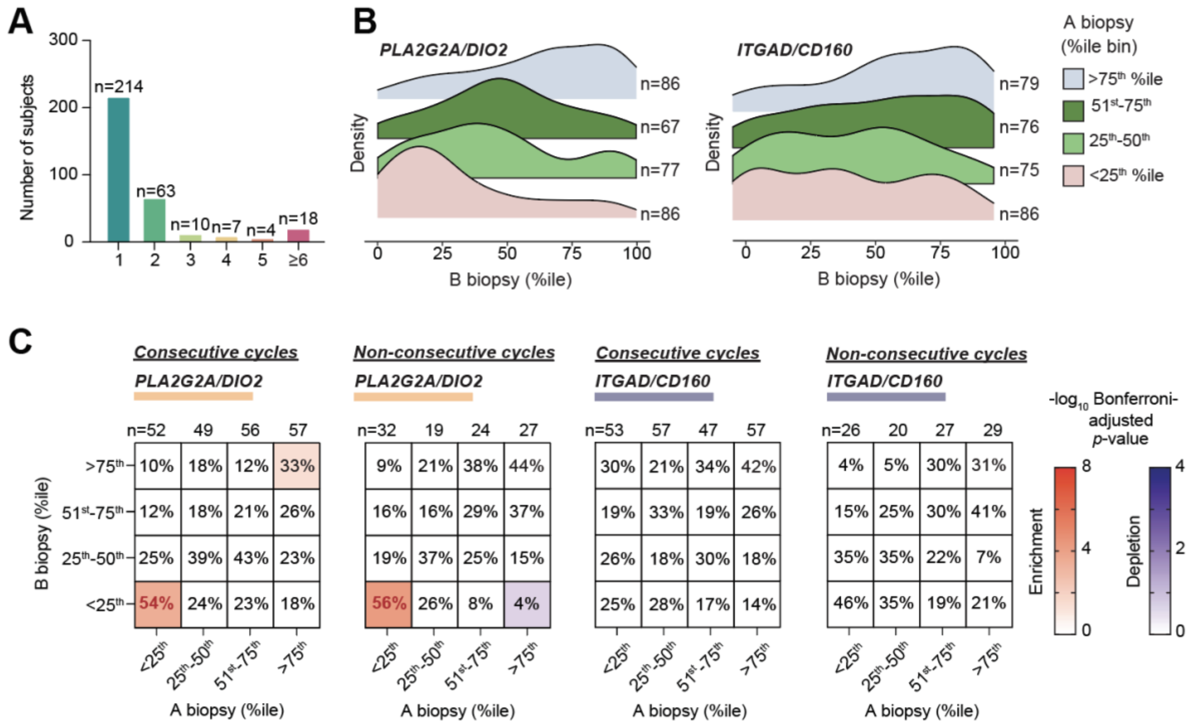

fig. S6. Analysis of intercycle variability. (**A**) Number of cycles between A and B biopsies. (**B**) Stacked histograms depicting the density of B-samples across the percentile range of normalized stromal and uNK subset ratios (left and right panel, respectively) for each corresponding A-sample quartile bin (color key). The number (n) of paired biopsies per A-sample quartile bin is indicated. The figure is a different representation of the data presented in Fig. 5B. (**C**) Contingency tables of the frequency of samples (%) grouped in quartile bins of normalized stromal and uNK subset ratios (*PLA2G2A/DIO2* %ile and *ITGAD/CD160* %ile, respectively) in paired A and B biopsies obtained in consecutive (n=214) or non-consecutive (n=102) cycles. The colored squares in the contingency tables indicate statistical significance ($p < 0.05$) as determined by the Fisher's exact test for enriched (red key) and depleted (blue key) associations.

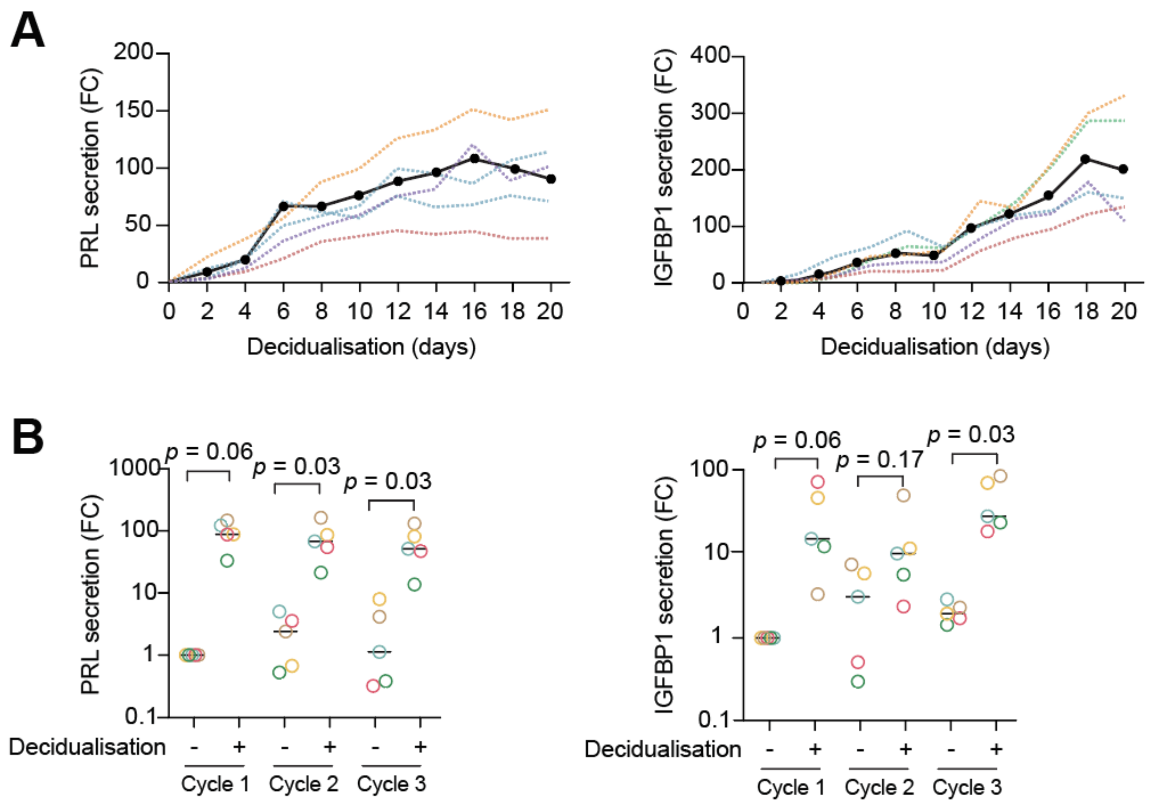

fig. S7. Prolactin (PRL) and insulin like growth factor binding protein 1 (IGFBP1) secretion by decidualizing assembloids. PRL and IGFBP1 are canonical decidual markers secreted by decidual and decidual-like senescent cells in culture. (**A**) Fold change (FC) in PRL and IGFBP1 secretion in endometrial assembloids decidualized for 20 days. Culture medium was refreshed every 48 hours. Colored dotted lines represent the FC in five biological repeat experiments. The solid black line represents the median FC. (**B**) Relative change in PRL and IGFBP1 secretion in assembloids across three 'cycles' of decidualization. Each 'cycle' comprised of four days of growth medium and four days of decidualization medium. Data show median FC in secreted levels in five color-coded biological repeat experiments. Statistical analysis is based on Wilcoxon matched-pairs signed rank test for comparison within each cycle.

Table S1. Subject demographics for bulk RNA-seq analysis of paired endometrial biopsies*.

Sample	Age (years)	LH+(days)	Prior live births (n=)	Prior miscarriages (n=)	BMI	Days between biopsies	*SCARA5*/*DIO2*** (%ile)
X17476	42	10	1	3	20	28	5
X17515		9					30
X17524	37	9	1	6	29.5	28	32
X17581		10					10
X19257	29	9	0	5	23	56	40
X19361		10					83
X17728	37	8	0	5	22	102	57
X18185		9					26
X17153	32	10	1	6	28	24	45
X17196		11					8
X19233	38	7	0	3	23	115	26
X19440		8					42

*Sample selection for this experiment was not based on demographic or clinical data.
***SCARA5*/*DIO2* %iles are based on a previously reported analysis of 250 timed endometrial samples (*19*).

Table S2. Subject demographics for functional assays*.

Assay	Figure	n=	**Age (years)** median (IQR)	**LH+(days)** median (IQR)	**Previous live births mean (range)**	**Previous miscarriages median (range)**	**BMI** median (IQR)
Single-cell RNA-seq	2F-H	4	36 (32.5-40.3)	5-6	0.25 (0-1)	3.5 (0-5)	25.8 (24-27.9)
		5	35 (32-39)	7-9	0 (0-0)	6 (2-13)	24.0 (23-25)
		3	38 (30-42)	10-11	0 (0-0)	3 (0-5)	29 (28-29)
uNK cell flow cytometry	2B-2C	6	39 (34-42)	6	0 (0-1)	3 (1-4)	25 (23-29.5)
		10	37.5 (32-40.25)	7	0 (0-2)	1 (0-5)	23 (22-27)
		12	36.5 (33-39.75)	8	0 (0-1)	4 (0-8)	23 (22-25.8)
		11	35 (32-40)	9	0 (0-1)	4 (0-8)	24 (23.25-29.8)
		12	34.5 (33-39.75)	10	0 (0-1)	4 (0-9)	25 (23.25-27)
		4	39.5 (32-45.5)	11	0 (0-1)	3 (0-4)	23 (21.25-27.8)
FACS (uNK subsets - killing assay)	2E	8	35 (34-38)	7 (5-7.8)	0.13 (0-1)	1.5 (1-10)	26 (21.8-29.8)
Primary stromal cell cultures		10	35.5 (33.5-36)	8.5 (8-9)	0.2 (0-1)	5 (1-5)	24 (23-27)
FACS (uNK subsets - purity)		11	38 (32.75-40)	8 (7-9)	0.1 (0-1)	1 (0-6)	26.5 (23.3-30)
Quantification of marker genes by ddPCR	2I-J	57	37 (34-38)	6	0 (0-3)	3 (0-17)	24 (21-26.5)
		179	35 (33-37)	7	0 (0-3)	3 (0-18)	24 (22-29)
		207	35 (32-38)	8	0 (0-4)	3 (0-14)	24 (21-27)
		202	34 (32-37)	9	0 (0-4)	3 (0-18)	24 (22-27)
		134	36 (33-38.5)	10	0 (0-3)	3 (0-15)	25 (22.6-28)
Spatial analysis of marker genes	3C, E, F & fig. S3A-C	264	36 (33-39)	8 (7-9)	0.18 (0-2)	3 (0-17)	24 (22-28)
Circulating hormone levels		315	36 (33-39)	8 (7-9)	0.16 (0-2)	2 (0-17)	24 (22-27.5)
Peripheral blood immune cells		94	36 (33-39)	8 (7-9)	0.19 (0-2)	3 (0-11)	24.24 (21.8-27)
T3 experiment	3H, K	11	35 (33-39)	8 (8-9)	0.1 (0-1)	3 (1-6)	22 (21-26)
Blastocyst conditioned media experiment		5	36 (34-39.5)	8 (7-8.25)	0.4 (0-1)	0 (0-7)	20 (20.7-31)
Prolonged decidualization	6C-G	5	33 (28-38.5)	8 (7-9.5)	0.2 (0-1)	5 (0-14)	24 (21-31)
Cyclical decidualization	6H-I	7	35.5 (35-37)	8.5 (6.5-10.25)	0.3 (0-1)	3 (1-7)	32 (27.5-35.5)

*Selection of samples for these experiments was not based on demographic or clinical data.

Table S3. Embryo grading and subject demographics.

Patient	Outcome	Age	BMI	Embryo grading	Procedure
1	Pregnant	31	24.4	BL4 AA	ICSI
2	Pregnant	35	20.7	BL4 AA	ICSI
3	Pregnant	33	21.3	BL5 AA	ICSI
4	Pregnant	29	21.3	BL4 AA	ICSI
5	Pregnant	30	26.1	BL4 AA	IVF
6	Pregnant	29	19	BL4 AA	IVF
7	Pregnant	26	33.9	C2	ICSI
8	Pregnant	34	25.5	BL4 AA	ICSI
9	Pregnant	30	24.7	BL4 BB	ICSI
10	Pregnant	31	27.9	BL4 BB	ICSI
11	Pregnant	38	27.2	BL4 CA	ICSI
12	Pregnant	27	22.2	BL5 AA	ICSI
13	Pregnant	29	19.3	BL3 AA	ICSI
14	Pregnant	38	33.3	BL4 BA	ICSI
15	Pregnant	37	24	BL4 AA	ICSI
16	Pregnant	19	29.9	BL4 AA	IVF
17	Pregnant	29	27.8	BL4 AA	IVF
18	Pregnant	32	21.3	BL4 BB	ICSI-ACC
19	Pregnant	33	19.4	BL4 BA	ICSI
20	Pregnant	37	24.5	BL4 BA	ICSI
21	Pregnant	31	22.4	BL4 AA	ICSI
22	Pregnant	40	27.8	BL4 AB	ICSI
23	Pregnant	26	19.1	BL4 BB	ICSI
24	Pregnant	33	20.9	BL4 BA	ICSI
25	Pregnant	38	25.1	BL4 BA	ICSI
26	Pregnant	32	23.1	BL4 AA	ICSI
27	Pregnant	39	23.3	BL4 AA	ICSI
28	Pregnant	39	25	BL4 BC	ICSI
29	Pregnant	35	23	BL4 BA	ICSI
30	Pregnant	31	18.7	BL4 AA	ICSI
31	Pregnant	37	21.6	BL4 AB	ICSI
32	Pregnant	27	22.6	BL4 AA	ICSI
33	Pregnant	30	19.9	BL4 AA	ICSI
34	Pregnant	39	25.8	BL4 AA	ICSI
35	Pregnant	34	23	BL4 AA	ICSI-ACC
36	Pregnant	40	24.2	BL4 AB	ICSI
37	Pregnant	34	20.1	BL3 BA	ICSI
38	Pregnant	32	22.9	BL4 AA	ICSI
39	Pregnant	40	22.4	BLA BA	ICSI

40	Pregnant	36	23.9	BL4 AA	ICSI
41	Not pregnant	29	23.8	BL4 AA	ICSI
42	Not pregnant	39	22.8	BL4 BB	ICSI
43	Not pregnant	35	27.3	BL4 AA	ICSI
44	Not pregnant	39	24.9	BL3 AC	ICSI
45	Not pregnant	34	20.5	BL2	ICSI
46	Not pregnant	30	28.1	BL4 BA	ICSI
47	Not pregnant	32	24.3	BL4 BB	ICSI
48	Not pregnant	27	22.8	BL3 AC	ICSI
49	Not pregnant	40	35.1	BL4 AB	ICSI
50	Not pregnant	41	29.3	BL4 BB	ICSI
51	Not pregnant	41	24	BL1	ICSI
52	Not pregnant	35	31.2	BL2	ICSI
53	Not pregnant	36	24.5	BL3 AB	ICSI
54	Not pregnant	30	22.3	BL4 AA	ICSI
55	Not pregnant	39	25.5	BL3 BB	ICSI
56	Not pregnant	39	27.5	BL4 BB & BL5 BA	ICSI
57	Not pregnant	43	25.6	BL4 AA	ICSI
58	Not pregnant	30	21.2	BL3 CB	IVF
59	Not pregnant	36	19.5	BL3 BA	IVF
60	Not pregnant	30	19	BL3 BB	ICSI
61	Not pregnant	40	26.4	BL4 AA	ICSI
62	Not pregnant	39	22.6	BL1	ICSI
63	Not pregnant	39	21.2	BL4 AA	ICSI
64	Not pregnant	31	20.7	BL4 BA	IVF
65	Not pregnant	35	29.6	BL2	ICSI
66	Not pregnant	42	24.1	BL3 BB	ICSI
67	Not pregnant	39	20.4	BL4 AA	ICSI
68	Not pregnant	35	23.7	BL4 AA	ICSI
69	Not pregnant	39	29.2	BL4 BA & BL4 BB	ICSI
70	Not pregnant	36	47.9	BL4 AA	ICSI
71	Not pregnant	40	27.8	BL2	ICSI
72	Not pregnant	33	31.7	BL4 AA	ICSI
73	Not pregnant	28	22.6	BL4 BA	ICSI
74	Not pregnant	39	28.4	BL4 BB	IVF
75	Not pregnant	37	19.3	BL4 AB	ICSI
76	Not pregnant	33	26.5	BL3 BB	IVF
77	Not pregnant	43	19.2	BL3 AB	ICSI
78	Not pregnant	34	32	BL5 BA	ICSI
79	Not pregnant	36	22.3	BL1	ICSI
80	Not pregnant	41	25.8	BL4 AA	ICSI

Group	Age (years) median (IQR)	BMI median (IQR)
**Pregnant**	33 (30-37)	23.05 (21.3-25.4)
**Non-pregnant**	36 (33-39)	24.4 (22.3-28.03)
***p*-value**	$p = 0.0032$	$p = 0.1258$

Data were tested for normality using Shapiro–Wilk test. Statistical significance was calculated by unpaired *t*-test (Age) or Mann-Whitney test (BMI)

Table S4. Subject demographics for retrospective cohort.

**Number of miscarriages** median (range)	**n=**	**Age (years)** median (IQR)	**LH+ (days)** median (IQR)	**BMI** median (IQR)	**Live births** mean [range]
0	162	35 (33-38)	8 (7-9)	23 (21-26)	0.09 [0-2]
1	99	36 (33-38)	8 (7-10)	24 (22-27)	0.11 [0-2]
2	92	36 (32-38.75)	8 (7-10)	24 (21-26.8)	0.12 [0-1]
3	88	35 (32-38)	8 (8-10)	24 (22-28)*	0.18 [0-3]
4	86	34.5 (32.8-36.3)	8 (7-9)	24 (22-27)	0.20 [0-2]
≥5 (5-18)	136	35 (32-38)	8 (7-10)	25 (23-29)*	0.33 [0-3]*

Data were tested for normality using Shapiro–Wilk test. Statistical significance was calculated by ANOVA with Dunnett's multiple comparison test for normally distributed data (age) or Kruskal-Wallis test with Dunn's multiple comparison test for non-normally distributed data (BMI, LH+, Previous miscarriages and live births) *denotes $p < 0.05$ compared to subjects with no previous pregnancy losses.

Table S5. Number of miscarriages and normalized circulating hormone levels - multilinear regression analysis

Number of previous losses	Unstandardized	Standardized	Standard error	t	*p*	95% confidence interval for B		ANOVA			
	Coefficients	Coefficients						Model	df	F	P
Model	B	Beta				lower bound	upper bound	Regression	3	3.44	0.017
Constant	2.08		0.44	4.72	<.001	1.21	2.96				
Progesterone (%ile)	0	-0.04	0.01	-0.71	0.476	-0.02	0.01				
Estradiol (%ile)	0	0	0.01	-0.01	0.995	-0.01	0.01				
TSH (%ile)	0.02	0.18	0.01	3.15	**0.002**	0.01	0.03				

Table S6. Marker gene ratios - multilinear regression analysis.

PLA2G2A/DIO2 (%ile)	Unstandardized	Standardized	Standard error	t	*p*	95% confidence interval for B		ANOVA			
	Coefficients	Coefficients						Model	df	F	*p*
**Model**	**B**	**Beta**				lower bound	upper bound	**Regression**	6	15.26	<.001
**Constant**	57.57		12.71	4.53	<.001	32.51	82.63				
**Number of previous losses**	-1.36	-0.13	0.42	-3.23	**0.001**	-2.19	-0.53				
**Age**	-0.3	-0.04	-1.11	-1.11	0.269	-0.82	0.23				
**Live births**	1.6	0.02	0.62	0.62	0.538	-3.52	6.72				
**BMI**	-0.48	-0.08	0.24	-1.97	0.05	-0.96	0				

ITGAD/CD160 (%ile)	Unstandardized	Standardized	Standard error	t	*p*	95% confidence interval for B		ANOVA			
	Coefficients	Coefficients						Model	df	F	p
**Model**	**B**	**Beta**				lower bound	upper bound	**Regression**	6	14.34	<.001
**Constant**	52.73		12.82	4.11	<.001	27.45	78				
**Number of previous losses**	-1.23	-0.12	0.42	-2.91	**0.004**	-2.07	-0.4				
**Age**	-0.06	-0.01	0.27	-0.24	0.811	-0.6	0.47				
**Live births**	-1.94	-0.03	2.61	-0.74	0.458	-7.09	3.21				
**BMI**	-0.29	-0.05	0.25	-1.18	0.238	-0.78	0.2				

Table S7. Subject demographics for prospective cohort

**Outcome**	**N=**	**Age (years)** median (IQR)	**LH+(days)** median (IQR)	**BMI** median (IQR)	**Live births** mean [range]	**Previous miscarriages** median [range]
Live birth	177	35 (31.3-37)	8 (7-9)	24.4 (22-29)	0.67 (0-5)	4 (0-14)
Miscarriage (all)	84	35 (33-37.5)	8 (7-9)	24.5 (22-30)	0.41 (0-3)	4 (1-15)
Miscarriage (unknown karyotype)	38	35 (33-37)	8 (7-9)	24.5 (22-30.3)	0.26 (0-1)	5 (1-15)
Miscarriage (euploid)	28	33 (32-37.8)	8.5 (7-10)	23.5 (22-27.8)	0.54 (0-3)	5 (2-14)
Miscarriage (aneuploid)	18	36.5 (33-39)	8 (7-9.3)	28 (23.2-30.8)	0.56 (0-3)	4 (3-14)

Table S8. Prospective cohort - logistic regression analysis.

All data						
	**Coefficient B**	**Standard error**	**z**	***p***	**Odds ratio**	**95% confidence interval**
**Constant**	2.44	1.78	1.37	0.169	11.51	0.35 - 376
**LH**	0.09	0.11	0.81	0.417	1.09	0.9 - 1.4
**Age**	-0.05	0.04	1.23	0.218	0.95	0.9 - 1.0
**Live births**	0.75	0.25	2.93	**0.003**	2.11	1.3 - 3.5
**Number of miscarriages**	-0.22	0.07	3.07	**0.002**	0.8	0.7 - 0.9
**BMI**	0	0.03	0.02	0.985	1	0.9 - 1.1

Table S9. Subject demographics for paired endometrial biopsy analysis

**Group** (number of previous miscarriage)	**n=**	**Previous miscarriages** median (range)	**Age (years)** median (IQR)	**LH+(days)** median (IQR)	**BMI** median (IQR)	**Live births** mean [range]	**Days between biopsies** median (IQR)
0	93	0	35 (32-39)	8 (7-9)	23 (21-26)	0.1 [0-2]	32 (28-56)
1-2	90	2 (1-2)	35 (32-39)	8 (7-9)	24.5 (21-28)	0.1 [0-2]	31 (28-57)
3-4	85	3 (3-4)	34 (32-36)	9 (7-9)	24 (22-28)	0.3 [0-2]*	31 (28-53)
≥5	48	5 (5-18)	35 (33-38)	8.5 (7-10)	27 (23-30)*	0.5 [0-4]*	28 (28-35)

Data were tested for normality using Shapiro–Wilk test. Statistical significance was calculated by ANOVA with Dunnett's multiple comparison test for normally distributed data (age) or Kruskal-Wallis test with Dunn's multiple comparison test for non-normally distributed data (BMI, LH+, previous miscarriages and live births). *denotes $p < 0.05$ compared to subjects with no previous pregnancy loss.

Table S10. Composition of assembloid culture media.

Expansion Medium (ExM)			
**Component**	**Manufacturer**	**Catalogue number**	**Final Concentration**
**Advanced DMEM/F12**	Life Technologies	12634010	1×
**N2**	Life Technologies	17502048	1×
**B27**	Life Technologies	12587010	1×
**Antibiotic-Antimycotic**	Gibco	15240062	1×
**L-glutamine**	Gibco	25030081	2 mM
**A83-01**	Merck Life Science	SML0788-5MG	500 nM
**R-spondin-1**	Peprotech	120-38	500 ng/mL
**Nicotinamide**	Merck Life Science	N0636	10 mM
**N-acetyl-L-cysteine**	Merck Life Science	A9165-5G	1.25 mM
**Noggin**	Peprotech	120-10c	100 ng/mL
**FGF10**	Peprotech	100-26	100 ng/mL
**HGF**	Peprotech	100-39	50 ng/mL
**EGF**	Peprotech	AF-100-15	50 ng/mL
**Minimal Decidualization Medium (MDM)**			
**Component**	**Manufacturer**	**Catalogue number**	**Final Concentration**
**Advanced DMEM/F12**	Life Technologies	12634010	1×
**N2**	Life Technologies	17502048	1×
**B27**	Life Technologies	12587010	1×
**Antibiotic-Antimycotic**	Gibco	15240062	1×
**L-glutamine**	Gibco	25030081	2 mM
**N-acetyl-L-cysteine**	Merck Life Science	A9165-5G	1.25 mM
**8-bromo-cAMP**	Merck Life Science	B7880	0.5 mM
**MPA**	Merck Life Science	M1629	1 µM
**β-estradiol**	Merck Life Science	E2758	1 nM

Table S11. Primer sequences for RT-qPCR.

Gene	Forward sequences (5'-3')	Reverse sequences (5'-3')
DIO2	ACT CGG TCA TTC TGC TCA A	TTC CAG ACG CAG CGC AGT
FOXO1	TGG ACA TGA TCA GCA GAC ATC	TTG GGT CAG GCG GTT CA
IL15	CAA ACA ACA GTT TGT CTT CT AAT GG	GGA CAA TAT GTA CAA AAC TCT GCA A
L19	GCG GAA GGG TAC AGC CAA	GCA GCC GGC GCA AA
MMP10	TTG ACC CCA ATG CCA GGA T	CCC CTA TCT CGC CTA GCA AT
PLA2G2A	AAA GGA AGC CGC ACT CAG TT	TTT CCA GGG AAG AGG GGA C
SCARA5	CAT GCG TGG GTT CAA AGG TG	CCA TTC ACC AGG CGG ATC AT

Table S12. Probes for ddPCR.

Assay name	Assay type	Bio-Rad assay ID	Probe fluorophore
CD160	Gene expression	dHsaCP2506689	HEX
DIO2	Gene expression	dHsaCNS365622194	HEX
ITGAD	Gene expression	dHsaCP2506311	FAM
PLA2G2A	Gene expression	dHsaCPE5042784	FAM

Table S13. Probes for smISH

Gene	Manufacturer	Catalogue number	Channel
DIO2	Bio-Techne LTD	562211	C2
PLA2G2A	Bio-Techne LTD	581101	C1

Table S14. Antibodies for immunohistochemistry.

Antibody	Company	Catalogue number	Sample	Host species	Antigen retrieval buffer	Dilution
CD56	Leica Biosystems	NCL-LCD56-504	Endometrial biopsy	Mouse	Tris-EDTA buffer, pH 9.0	1:100 in TBST
KIR2DL1/L2/L3/S1/S2	Abcam	ab252938	Endometrial biopsy	Rabbit	Tris-EDTA buffer, pH 9.0	1:1000 in TBST
E-cadherin	Cell Signalling Technology	24E10	Endometrial biopsy/explant culture	Rabbit	Sodium citrate buffer, pH 6.0	1:200 0.05% TBS-T
Vimentin	Cell Signalling Technology	5G3F10	Endometrial biopsy/explant culture	Mouse	Sodium citrate buffer, pH 6.0	1:200 0.05%TBST
E-cadherin	Cell Signalling Technology	24E10	IHC for assembloids	Rabbit	NA	1:200 10%FBS-2%BSA-PBS-0.05%Tween-20
Vimentin	Cell Signalling Technology	5G3F10	IHC for assembloids	Mouse	NA	1:200 10%FBS-2%BSA-PBS-0.05%Tween-20
Goat anti-Rabbit IgG Highly Cross-Absorbed Secondary Antibody, Alexa Fluor™ 647	Invitrogen	A21245	IHC for assembloids	Goat	NA	1:1000 in 10%FBS-2%BSA-PBS-0.05%Tween-20
Donkey anti-Mouse IgG Highly Cross-Absorbed Secondary Antibody, Alexa Fluor™ 594	Invitrogen	A21203	IHC for assembloids	Donkey	NA	1:1000 in 10%FBS-2%BSA-PBS-0.05%Tween-20
Donkey anti-Rabbit IgG (H+L) Highly Cross-Adsorbed Secondary Antibody, Alexa Fluor™ 488	Invitrogen	A21206	Endometrial biopsy/explant culture	Donkey	NA	1:1000 in 0.05% TBS-T
Donkey anti-Mouse IgG Highly Cross-Absorbed Secondary Antibody, Alexa Fluor™ 594	Invitrogen	A21203	Endometrial biopsy/explant culture	Donkey	NA	1:1000 in 0.05% TBS-T

Table S15. Antibodies for flow cytometry and sorting of primary uNK cells.

Antibody	Manufacturer	Catalogue number	Sample	Fluorophore	Clone	Dilution
Fixable Viability Stain 660	BD Biosciences	564405	Endometrial tissue	APC	NA	1:1000 in PBS
CD45	BioLegend	304006	Endometrial tissue	FITC	HI30	1:40 in 0.5%BSA/PBS
CD49a	BioLegend	328312	Endometrial tissue	PE-Cy7	TS2/7	1:40 in 0.5%BSA/PBS
CD158d (KI2DL4)	BioLegend	347006	Endometrial tissue	PE	MaB 33	1:40 in 0.5%BSA/PBS
CD158b (KIR2DL1/S1/S3/S5)	BioLegend	339506	Endometrial tissue	PE	HP-MA4	1:40 in 0.5%BSA/PBS
CD158b (KIR2DL2/DL3)	Miltenyi Biotec	130-092-618	Endometrial tissue	PE	DX27	1:20 in 0.5%BSA/PBS
CD3	BD Biosciences	644611	Blood	FITC	SK7	20µL antibody mixture/50µL blood
CD16	BD Biosciences	644611	Blood	PE	B73.1	20µL antibody mixture/50µL blood
CD45	BD Biosciences	644611	Blood	PerCP-Cy5.5	2D1	20µL antibody mixture/50µL blood
CD56	BD Biosciences	644611	Blood	PE	NCAM16.2	20µL antibody mixture/50µL blood

Other Supplementary Material for this manuscript includes:
Date file S1: Bulk and single-cell RNA-seq analysis and receptor-ligand predictions.
Date file S2: Source files

REFERENCES AND NOTES

1. S. Quenby, I. D. Gallos, R. K. Dhillon-Smith, M. Podesek, M. D. Stephenson, J. Fisher, J. J. Brosens, J. Brewin, R. Ramhorst, E. S. Lucas, R. C. McCoy, R. Anderson, S. Daher, L. Regan, M. al-Memar, T. Bourne, D. A. MacIntyre, R. Rai, O. B. Christiansen, M. Sugiura-Ogasawara, J. Odendaal, A. J. Devall, P. R. Bennett, S. Petrou, A. Coomarasamy, Miscarriage matters: The epidemiological, physical, psychological, and economic costs of early pregnancy loss. *Lancet* **397**, 1658–1667 (2021).

2. J. Muter, V. J. Lynch, R. C. McCoy, J. J. Brosens, Human embryo implantation. *Development* **150**, dev201507 (2023).

3. J. J. Brosens, P. R. Bennett, V. M. Abrahams, R. Ramhorst, A. Coomarasamy, S. Quenby, E. S. Lucas, R. C. McCoy, Maternal selection of human embryos in early gestation: Insights from recurrent miscarriage. *Semin. Cell Dev. Biol.* **131**, 14–24 (2022).

4. R. C. McCoy, Mosaicism in preimplantation human embryos: When chromosomal abnormalities are the norm. *Trends Genet.* **33**, 448–463 (2017).

5. B. Gellersen, J. J. Brosens, Cyclic decidualization of the human endometrium in reproductive health and failure. *Endocr. Rev.* **35**, 851–905 (2014).

6. N. S. Macklon, J. J. Brosens, The human endometrium as a sensor of embryo quality. *Biol. Reprod.* **91**, 98 (2014).

7. M. C. Magnus, A. J. Wilcox, N. H. Morken, C. R. Weinberg, S. E. Håberg, Role of maternal age and pregnancy history in risk of miscarriage: Prospective register based study. *BMJ* **364**, l869 (2019).

8. A. M. Kolte, D. Westergaard, O. Lidegaard, S. Brunak, H. S. Nielsen, Chance of live birth: A nationwide, registry-based cohort study. *Hum. Reprod.* **36**, 1065–1073 (2021).

9. H. A. Risch, N. S. Weiss, E. A. Clarke, A. B. Miller, Risk factors for spontaneous abortion and its recurrence. *Am. J. Epidemiol.* **128**, 420–430 (1988).

10. A. Webster, M. Schuh, Mechanisms of aneuploidy in human eggs. *Trends Cell Biol.* **27**, 55–68 (2017).

11. K. Hardy, P. J. Hardy, P. A. Jacobs, K. Lewallen, T. J. Hassold, Temporal changes in chromosome abnormalities in human spontaneous abortions: Results of 40 years of analysis. *Am. J. Med. Genet. A* **170**, 2671–2680 (2016).

12. N. J. Raine-Fenning, B. K. Campbell, J. S. Clewes, N. R. Kendall, I. R. Johnson, Defining endometrial growth during the menstrual cycle with three-dimensional ultrasound. *BJOG* **111**, 944–949 (2004).

13. W. Wang, F. Vilella, P. Alama, I. Moreno, M. Mignardi, A. Isakova, W. Pan, C. Simon, S. R. Quake, Single-cell transcriptomic atlas of the human endometrium during the menstrual cycle. *Nat. Med.* **26**, 1644–1653 (2020).

14. R. W. Noyes, A. T. Hertig, J. Rock, Dating the endometrial biopsy. *Am. J. Obstet. Gynecol.* **122**, 262–263 (1975).

15. B. Strunz, J. Bister, H. Jönsson, I. Filipovic, Y. Crona-Guterstam, E. Kvedaraite, N. Sleiers, B. Dumitrescu, M. Brännström, A. Lentini, B. Reinius, M. Cornillet, T. Willinger, S. Gidlöf, R. S. Hamilton, M. A. Ivarsson, N. K. Björkström, Continuous human uterine NK cell differentiation in response to endometrial regeneration and pregnancy. *Sci. Immunol.* **6**, eabb7800 (2021).

16. A. R. Chavan, O. W. Griffith, D. J. Stadtmauer, J. Maziarz, M. Pavlicev, R. Fishman, L. Koren, R. Romero, G. P. Wagner, Evolution of embryo implantation was enabled by the origin of decidual stromal cells in eutherian mammals. *Mol. Biol. Evol.* **38**, 1060–1074 (2021).

17. P. Vrljicak, E. S. Lucas, M. Tryfonos, J. Muter, S. Ott, J. J. Brosens, Dynamic chromatin remodeling in cycling human endometrium at single-cell level. *Cell Rep.* **42**, 113525 (2023).

18. P. J. Brighton, Y. Maruyama, K. Fishwick, P. Vrljicak, S. Tewary, R. Fujihara, J. Muter, E. S. Lucas, T. Yamada, L. Woods, R. Lucciola, Y. Hou Lee, S. Takeda, S. Ott, M. Hemberger, S.

Quenby, J. J. Brosens, Clearance of senescent decidual cells by uterine natural killer cells in cycling human endometrium. *eLife* **6**, e31274 (2017).

19. E. S. Lucas, P. Vrljicak, J. Muter, M. M. Diniz-da-Costa, P. J. Brighton, C. S. Kong, J. Lipecki, K. J. Fishwick, J. Odendaal, L. J. Ewington, S. Quenby, S. Ott, J. J. Brosens, Recurrent pregnancy loss is associated with a pro-senescent decidual response during the peri-implantation window. *Commun. Biol.* **3**, 37 (2020).

20. A. T. Hertig, J. Rock, E. C. Adams, A description of 34 human ova within the first 17 days of development. *Am. J. Anat.* **98**, 435–493 (1956).

21. T. M. Rawlings, K. Makwana, D. M. Taylor, M. A. Molè, K. J. Fishwick, M. Tryfonos, J. Odendaal, A. Hawkes, M. Zernicka-Goetz, G. M. Hartshorne, J. J. Brosens, E. S. Lucas, Modelling the impact of decidual senescence on embryo implantation in human endometrial assembloids. *eLife* **10**, e69603 (2021).

22. G. Lambeau, M. H. Gelb, Biochemistry and physiology of mammalian secreted phospholipases A_2. *Annu. Rev. Biochem.* **77**, 495–520 (2008).

23. D. J. Stadtmauer, G. P. Wagner, Single-cell analysis of prostaglandin E_2-induced human decidual cell in vitro differentiation: A minimal ancestral deciduogenic signaldagger. *Biol. Reprod.* **106**, 155–172 (2022).

24. M. De Cecco, S. W. Criscione, E. J. Peckham, S. Hillenmeyer, E. A. Hamm, J. Manivannan, A. L. Peterson, J. A. Kreiling, N. Neretti, J. M. Sedivy, Genomes of replicatively senescent cells undergo global epigenetic changes leading to gene silencing and activation of transposable elements. *Aging Cell* **12**, 247–256 (2013).

25. A. Freund, A. V. Orjalo, P. Y. Desprez, J. Campisi, Inflammatory networks during cellular senescence: Causes and consequences. *Trends Mol. Med.* **16**, 238–246 (2010).

26. R. Mullur, Y. Y. Liu, G. A. Brent, Thyroid hormone regulation of metabolism. *Physiol. Rev.* **94**, 355–382 (2014).

27. M. Mericskay, J. Kitajewski, D. Sassoon, *Wnt5a* is required for proper epithelial-mesenchymal interactions in the uterus. *Development* **131**, 2061–2072 (2004).

28. M. A. Nieto, R. Y. Huang, R. A. Jackson, J. P. Thiery, EMT: 2016. *Cell* **166**, 21–45 (2016).

29. A. Moffett, N. Shreeve, Local immune recognition of trophoblast in early human pregnancy: Controversies and questions. *Nat. Rev. Immunol.* **23**, 222–235 (2023).

30. A. Erlebacher, Immunology of the maternal-fetal interface. *Annu. Rev. Immunol.* **31**, 387–411 (2013).

31. L. Catalini, J. Fedder, Characteristics of the endometrium in menstruating species: Lessons learned from the animal kingdom. *Biol. Reprod.* **102**, 1160–1169 (2020).

32. E. Vivier, E. Tomasello, M. Baratin, T. Walzer, S. Ugolini, Functions of natural killer cells. *Nat. Immunol.* **9**, 503–510 (2008).

33. F. Wang, W. Jia, M. Fan, X. Shao, Z. Li, Y. Liu, Y. Ma, Y. X. Li, R. Li, Q. Tu, Y. L. Wang, Single-cell immune landscape of human recurrent miscarriage. *Genom. Proteom. Bioinform.* **19**, 208–222 (2021).

34. C. Guo, P. Cai, L. Jin, Q. Sha, Q. Yu, W. Zhang, C. Jiang, Q. Liu, D. Zong, K. Li, J. Fang, F. Lu, Y. Wang, D. Li, J. Lin, L. Li, Z. Zeng, X. Tong, H. Wei, K. Qu, Single-cell profiling of the human decidual immune microenvironment in patients with recurrent pregnancy loss. *Cell Discov.* **7**, 1 (2021).

35. P. Sharma, S. Thakran, X. Deng, M. B. Elam, E. A. Park, Nuclear corepressors mediate the repression of phospholipase A_2 group IIa gene transcription by thyroid hormone. *J. Biol. Chem.* **288**, 16321–16333 (2013).

36. C. L. Lee, M. Vijayan, X. Wang, K. K. W. Lam, H. Koistinen, M. Seppala, R. H. W. Li, E. H. Y. Ng, W. S. B. Yeung, P. C. N. Chiu, Glycodelin-A stimulates the conversion of human peripheral blood CD16-$CD56^{bright}$ NK cell to a decidual NK cell-like phenotype. *Hum. Reprod.* **34**, 689–701 (2019).

37. L. Mosteiro, C. Pantoja, N. Alcazar, R. M. Marión, D. Chondronasiou, M. Rovira, P. J. Fernandez-Marcos, M. Muñoz-Martin, C. Blanco-Aparicio, J. Pastor, G. Gómez-López, A. de Martino, M. A. Blasco, M. Abad, M. Serrano, Tissue damage and senescence provide critical signals for cellular reprogramming in vivo. *Science* **354**, (2016).

38. R. A. Shivdasani, H. Clevers, F. J. de Sauvage, Tissue regeneration: Reserve or reverse? *Science* **371**, 784–786 (2021).

39. D. Munoz-Espin, M. Serrano, Cellular senescence: From physiology to pathology. *Nat. Rev. Mol. Cell Biol.* **15**, 482–496 (2014).

40. S. Zeng, Y. Liang, S. Lai, S. Bi, L. Huang, Y. Li, W. Deng, P. Xu, M. Liu, Z. Xiong, J. Chen, Z. Tu, D. Chen, L. Du, TNFα/TNFR1 signal induces excessive senescence of decidua stromal cells in recurrent pregnancy loss. *J. Reprod. Immunol.* **155**, 103776 (2023).

41. H. P. Gaide Chevronnay, C. Selvais, H. Emonard, C. Galant, E. Marbaix, P. Henriet, Regulation of matrix metalloproteinases activity studied in human endometrium as a paradigm of cyclic tissue breakdown and regeneration. *Biochim. Biophys. Acta* **1824**, 146–156 (2012).

42. M. L. Meizlish, R. A. Franklin, X. Zhou, R. Medzhitov, Tissue homeostasis and inflammation. *Annu. Rev. Immunol.* **39**, 557–581 (2021).

43. M. Schewe, P. F. Franken, A. Sacchetti, M. Schmitt, R. Joosten, R. Böttcher, M. E. van Royen, L. Jeammet, C. Payré, P. M. Scott, N. R. Webb, M. Gelb, R. T. Cormier, G. Lambeau, R. Fodde, Secreted phospholipases A2 are intestinal stem cell niche factors with distinct roles in homeostasis, inflammation, and cancer. *Cell Stem Cell* **19**, 38–51 (2016).

44. X. Fan, S. Krieg, J. Y. Hwang, S. Dhal, C. J. Kuo, B. L. Lasley, R. M. Brenner, N. R. Nayak, Dynamic regulation of Wnt7a expression in the primate endometrium: Implications for postmenstrual regeneration and secretory transformation. *Endocrinology* **153**, 1063–1069 (2012).

45. R. P. Berkhout, C. B. Lambalk, S. Repping, G. Hamer, S. Mastenbroek, Premature expression of the decidualization marker prolactin is associated with repeated implantation failure. *Gynecol. Endocrinol.* **36**, 360–364 (2020).

46. A. J. Wilcox, D. D. Baird, C. R. Weinberg, Time of implantation of the conceptus and loss of pregnancy. *N. Engl. J. Med.* **340**, 1796–1799 (1999).

47. S. Perdu, B. Castellana, Y. Kim, K. Chan, L. DeLuca, A. G. Beristain, Maternal obesity drives functional alterations in uterine NK cells. *JCI Insight* **1**, e85560 (2016).

48. ESHRE Guideline Group on RPL, R. B. Atik, O. B. Christiansen, J. Elson, A. M. Kolte, S. Lewis, S. Middeldorp, S. Mcheik, B. Peramo, S. Quenby, H. S. Nielsen, M.-L. van der Hoorn, N. Vermeulen, M. Goddijn, ESHRE guideline: Recurrent pregnancy loss: An update in 2022. *Hum. Reprod. Open* **2023**, hoad002 (2023).

49. M. C. Magnus, N. H. Morken, K. A. Wensaas, A. J. Wilcox, S. E. Haberg, Risk of miscarriage in women with chronic diseases in Norway: A registry linkage study. *PLoS Med.* **18**, e1003603 (2021).

50. M. C. Magnus, R. L. Hockey, S. E. Haberg, G. D. Mishra, Pre-pregnancy lifestyle characteristics and risk of miscarriage: The Australian Longitudinal Study on Women's Health. *BMC Pregnancy Childbirth* **22**, 169 (2022).

51. E. S. Lucas, N. P. Dyer, K. Murakami, Y. H. Lee, Y. W. Chan, G. Grimaldi, J. Muter, P. J. Brighton, J. D. Moore, G. Patel, J. K. Y. Chan, S. Takeda, E. W. F. Lam, S. Quenby, S. Ott, J. J. Brosens, Loss of endometrial plasticity in recurrent pregnancy loss. *Stem Cells* **34**, 346–356 (2016).

52. S. Gordts, G. Grimbizis, V. Tanos, P. Koninckx, R. Campo, Junctional zone thickening: An endo-myometrial unit disorder. *Facts Views Vis. Obgyn* **15**, 309–316 (2023).

53. A. Busnelli, G. Barbaro, F. Pozzati, S. D'Ippolito, M. Cristodoro, E. Nobili, G. Scambia, N. di Simone, The importance of the 'uterine factor' in recurrent pregnancy loss: A retrospective

cohort study on women screened through 3D transvaginal ultrasound. *Hum. Reprod.* **39**, 1645–1655 (2024).

54. M. Cozzolino, M. Cosentino, L. Loiudice, F. G. Martire, D. Galliano, A. Pellicer, C. Exacoustos, Impact of adenomyosis on in vitro fertilization outcomes in women undergoing donor oocyte transfers: A prospective observational study. *Fertil. Steril.* **121**, 480–488 (2024).

55. A. M. Kolte, L. A. Bernardi, O. B. Christiansen, S. Quenby, R. G. Farquharson, M. Goddijn, M. D. Stephenson, ESHRE Special Interest Group, Early Pregnancy, Terminology for pregnancy loss prior to viability: A consensus statement from the ESHRE early pregnancy special interest group. *Hum. Reprod.* **30**, 495–498 (2015).

56. J. Odendaal, N. Black, P. R. Bennett, J. Brosens, S. Quenby, D. A. MacIntyre, The endometrial microbiota and early pregnancy loss. *Hum. Reprod.* **39**, 638–646 (2024).

57. A. Coomarasamy, A. J. Devall, J. J. Brosens, S. Quenby, M. D. Stephenson, S. Sierra, O. B. Christiansen, R. Small, J. Brewin, T. E. Roberts, R. Dhillon-Smith, H. Harb, H. Noordali, A. Papadopoulou, A. Eapen, M. Prior, G. C. di Renzo, K. Hinshaw, B. W. Mol, M. A. Lumsden, Y. Khalaf, A. Shennan, M. Goddijn, M. van Wely, M. al-Memar, P. Bennett, T. Bourne, R. Rai, L. Regan, I. D. Gallos, Micronized vaginal progesterone to prevent miscarriage: A critical evaluation of randomized evidence. *Am. J. Obstet. Gynecol.* **223**, 167–176 (2020).

58. T. S. Hartwig, L. Ambye, J. R. Gruhn, J. F. Petersen, T. Wrønding, L. Amato, A. C.-H. Chan, B. Ji, M. H. Bro-Jørgensen, L. Werge, M. M. B. S. Petersen, C. Brinkmann, J. B. Ribberholt, M. Dunø, I. Bache, M. J. Herrgård, F. S. Jørgensen, E. R. Hoffmann, H. S. Nielsen, COPL consortium, Cell-free fetal DNA for genetic evaluation in Copenhagen Pregnancy Loss Study (COPL): A prospective cohort study. *Lancet* **401**, 762–771 (2023).

59. R. K. Dhillon-Smith, P. Melo, A. J. Devall, P. P. Smith, M. al-Memar, K. Barnhart, G. Condous, O. B. Christiansen, M. Goddijn, D. Jurkovic, D. Lissauer, A. Maheshwari, O. T. Oladapo, J. Preisler, L. Regan, R. Small, M. Stephenson, C. Wijeyaratne, S. Quenby, T. Bourne, A. Coomarasamy, A core outcome set for trials in miscarriage management and prevention: An international consensus development study. *BJOG* **130**, 1346–1354 (2023).

60. O. Khan, S. Gour, S. N. L. C. Keung, N. Morris, R. Shields, S. Quenby, D. B. Dimakou, O. Pickering, J. Tamblyn, A. Devall, A. Coomarasamy, D. K. Thornton, A. Perry, T. N. Arvanitis, Electronic patient reported outcomes for miscarriage research in Tommy's net. *Stud. Health Technol. Inform.* **295**, 458–461 (2022).

61. J. C. Prior, M. Naess, A. Langhammer, S. Forsmo, Ovulation prevalence in women with spontaneous normal-length menstrual cycles—A population-based cohort from HUNT3, Norway. *PLOS ONE* **10**, e0134473 (2015).

62. W. N. Venables, B. D. Ripley, *Modern Applied Statistics with S* (Springer, ed. 4, 2002).

63. M. L. Delignette-Muller, C. Dutang, fitdistrplus: An R package for fitting distributions. *J. Stat. Softw.* **64**, 1–34 (2015).

64. H. Van de Velde, A. De Vos, H. Joris, Z. P. Nagy, A. C. Van Steirteghem, Effect of timing of oocyte denudation and micro-injection on survival, fertilization and embryo quality after intracytoplasmic sperm injection. *Hum. Reprod.* **13**, 3160–3164 (1998).

65. P. Platteau, E. Laurent, C. Albano, K. Osmanagaoglu, V. Vernaeve, H. Tournaye, M. Camus, A. van Steirteghem, P. Devroey, An open, randomized single-centre study to compare the efficacy and convenience of follitropin beta administered by a pen device with follitropin alpha administered by a conventional syringe in women undergoing ovarian stimulation for IVF/ICSI. *Hum. Reprod.* **18**, 1200–1204 (2003).

66. D. K. Gardner, W. B. Schoolcraft, Culture and transfer of human blastocysts. *Curr. Opin. Obstet. Gynecol.* **11**, 307–311 (1999).

67. A. Dobin, C. A. Davis, F. Schlesinger, J. Drenkow, C. Zaleski, S. Jha, P. Batut, M. Chaisson, T. R. Gingeras, STAR: Ultrafast universal RNA-seq aligner. *Bioinformatics* **29**, 15–21 (2013).

68. M. I. Love, W. Huber, S. Anders, Moderated estimation of fold change and dispersion for RNA-seq data with DESeq2. *Genome Biol.* **15**, 550 (2014).

69. M. D. Young, S. Behjati, SoupX removes ambient RNA contamination from droplet-based single-cell RNA sequencing data. *Gigascience* **9**, giaa151 (2020).

70. A. Butler, P. Hoffman, P. Smibert, E. Papalexi, R. Satija, Integrating single-cell transcriptomic data across different conditions, technologies, and species. *Nat. Biotechnol.* **36**, 411–420 (2018).

71. C. S. McGinnis, L. M. Murrow, Z. J. Gartner, DoubletFinder: Doublet detection in single-cell RNA sequencing data using artificial nearest neighbors. *Cell Syst* **8**, 329–337.e4 (2019).

72. S. Marsh, scCustomize: Custom visualizations & functions for streamlined analyses of single cell sequencing (2021).

73. A. Subramanian, P. Tamayo, V. K. Mootha, S. Mukherjee, B. L. Ebert, M. A. Gillette, A. Paulovich, S. L. Pomeroy, T. R. Golub, E. S. Lander, J. P. Mesirov, Gene set enrichment analysis: A knowledge-based approach for interpreting genome-wide expression profiles. *Proc. Natl. Acad. Sci. U.S.A.* **102**, 15545–15550 (2005).

74. M. Efremova, M. Vento-Tormo, S. A. Teichmann, R. Vento-Tormo, CellPhoneDB: Inferring cell-cell communication from combined expression of multi-subunit ligand-receptor complexes. *Nat. Protoc.* **15**, 1484–1506 (2020).

75. S. Petropoulos, D. Edsgärd, B. Reinius, Q. Deng, S. P. Panula, S. Codeluppi, A. P. Reyes, S. Linnarsson, R. Sandberg, F. Lanner, Single-cell RNA-seq reveals lineage and X chromosome dynamics in human preimplantation embryos. *Cell* **167**, 285 (2016).

76. G. L. Manno, R. Soldatov, A. Zeisel, E. Braun, H. Hochgerner, V. Petukhov, K. Lidschreiber, M. E. Kastriti, P. Lönnerberg, A. Furlan, J. Fan, L. E. Borm, Z. Liu, D. van Bruggen, J. Guo, X. He, R. Barker, E. Sundström, G. Castelo-Branco, P. Cramer, I. Adameyko, S. Linnarsson, P. V. Kharchenko, RNA velocity of single cells. *Nature* **560**, 494–498 (2018).

77. V. Bergen, M. Lange, S. Peidli, F. A. Wolf, F. J. Theis, Generalizing RNA velocity to transient cell states through dynamical modeling. *Nat. Biotechnol.* **38**, 1408–1414 (2020).

78. R. Rodriguez-Quiroz, B. Valdebenito-Maturana, SoloTE for improved analysis of transposable elements in single-cell RNA-Seq data using locus-specific expression. *Commun. Biol.* **5**, 1063 (2022).

79. C. A. Schneider, W. S. Rasband, K. W. Eliceiri, NIH Image to ImageJ: 25 years of image analysis. *Nat. Methods* **9**, 671–675 (2012).

80. S. Preibisch, S. Saalfeld, P. Tomancak, Globally optimal stitching of tiled 3D microscopic image acquisitions. *Bioinformatics* **25**, 1463–1465 (2009).
